# A Hitchhiker’s guide to the potato wart disease resistance galaxy

**DOI:** 10.1007/s00122-020-03678-x

**Published:** 2020-09-12

**Authors:** Charlotte Prodhomme, Gert van Arkel, Jarosław Plich, Jasper E. Tammes, Johan Rijk, Herman J. van Eck, Richard G. F. Visser, Jack H. Vossen

**Affiliations:** 1 Plant Breeding, Wageningen University and Research, P.O. Box 386, 6700 AJ Wageningen, The Netherlands; 2 Plant Breeding and Acclimatization Institute – National Research Institute (IHAR‑PIB), Młochów Research Center, Platanowa 19, 05‑831 Młochów, Poland; 3 Present Address: Averis Seeds B.V., Valtherblokken Z40, 7876 TC Valthermond, The Netherlands; 4 Present Address: Pop Vriend Seeds B.V., Middenweg 52, 1619 BN Andijk, The Netherlands; 5 Present Address: FN3PT/Inov3PT, INRAE Keraiber, 29260 Ploudaniel, France

## Abstract

**Key message:**

Two novel major effect loci (*Sen4* and *Sen5*) and several minor effect QTLs for potato wart disease resistance have been mapped. The importance of minor effect loci to bring full resistance to wart disease was investigated. Using the newly identified and known wart disease resistances, a panel of potato breeding germplasm and Solanum wild species was screened. This provided a state-of-the-art “hitch-hikers-guide” of complementary wart disease resistance sources.

**Abstract:**

Potato wart disease, caused by the obligate biotrophic soil-born fungus *Synchytrium endobioticum*, is the most important quarantine disease of potato. Because of its huge impact on yield, the lack of chemical control and the formation of resting spores with long viability, breeding for resistant varieties combined with strict quarantine measures are the only way to efficiently and durably manage the disease. In this study, we set out to make an inventory of the different resistance sources. Using a Genome-Wide Association Study (GWAS) in the potato breeding genepool, we identified *Sen4*, associated with pathotypes 2, 6 and 18 resistance. Associated SNPs mapped to the south arm of chromosome *12* and were validated to be linked to resistance in one full-sib population. Also, a bulked segregant analysis combined with a Comparative Subsequence Sets Analysis (CoSSA) resulted in the identification of *Sen5*, associated with pathotypes 2, 6 and 18 resistance, on the south arm of chromosome *5.* In addition to these two major effect loci, the GWAS and CoSSA allowed the identification of several quantitative trait loci necessary to bring full resistance to certain pathotypes. Panels of varieties and *Solanum* accessions were screened for the presence of *Sen1*, *Sen2*, *Sen3*, *Sen4* and *Sen5.* Combined with pedigree analysis, we could trace back some of these genes to the ancestral resistance donors. This analysis revealed complementary resistance sources and allows elimination of redundancy in wart resistance breeding programs.

**Electronic supplementary material:**

The online version of this article (10.1007/s00122-020-03678-x) contains supplementary material, which is available to authorized users.

## Introduction

Every year, food crop production suffers huge yield losses due to pests and pathogen attacks. For potato (*Solanum tuberosum*), the yield losses have been estimated between 8 and 21% depending on the region of production (Savary et al. 2019). The potato wart disease, caused by *Synchytrium endobioticum*, can cause yield losses up to 100%. *S. endobioticum* is an obligate biotrophic soil-born fungus from the Chytridiomycota phylum which causes the formation of galls on potato tubers. After the sexual phase of its life cycle, *S. endobioticum* produces winter spores (Curtis 1921) which can remain viable in the soil for decades (Przetakiewicz 2015). Because of this longevity and the lack of efficient chemical control (Hampson 1988), *S. endobioticum* has a quarantine status worldwide. The fungus originates from the Andean region of South America where it co-evolved with potato species and was introduced in North America and Europe at the end of the nineteenth century (Hampson and Proudfoot 1974). At that time, only one variant (pathotype 1 (D1)) of the pathogen existed and breeders were successful in breeding for resistant varieties at the beginning of the twentieth century. However, new pathotypes emerged in the 1940s (Maris 1961) and resistance to the pathotype 1 was not effective against them. Today, more than 40 pathotypes of *S. endobioticum* have been reported and the most frequent occurring in Europe are pathotypes 1(D1), 2(G1), 6(O1) and 18(T1). There is a strong need of identifying loci bringing resistance against these higher pathotypes as the only efficient and durable way to control the potato wart disease is to apply strict quarantine measures and cultivate resistant potato varieties.

Several potato wart disease resistance genes have already been identified. The first gene to be identified was *Sen1* (Hehl et al. 1999), a TIR-NBS-LRR (TNL) gene from the chromosome *11* cluster C76 (Prodhomme et al. 2020a), which brings resistance to pathotype 1 through the recognition of the pathogen effector AvrSen1 (van de Vossenberg et al. 2019). *Sen1* has been intensively used in breeding (Prodhomme et al. 2020a), and its resistance is not effective against higher pathotypes. The *Sen2* gene, bringing resistance to a wide range of pathotypes, has been identified recently in a complex diploid species hybrid. Its presence in commercial varieties is unknown as the markers flanking *Sen2* have not yet been screened in a panel of resistant and susceptible varieties (Plich et al. 2018). *Sen3*, bringing resistance to pathotypes 2, 6 and 18, has been mapped to the same TNL cluster as *Sen1* (Prodhomme et al. 2019). The flanking markers of *Sen3* have been screened in a wider panel of varieties (Bartkiewicz et al. 2018; Prodhomme et al. 2019) and *Sen3* was found to be the main cause of resistance in Polish and German varieties. These three *Sen* genes are dominant genes giving a qualitative type of resistance. Several quantitative trait loci (QTLs) have also been mapped in tetraploid populations. In the BNA1 and SaKa1 tetraploid populations, *Sen1* was identified as well as a QTL on chromosome *1* giving resistance to pathotypes 2, 6 and 18 and a QTL on chromosome *9* giving resistance to pathotype 18 (Ballvora et al. 2011). In the full-sib population obtained from the cross between Saturna and Panda, *Sen1* was identified, as well as several QTLs on chromosomes *1*, *2*, *6*, *7*, *8*, *10* and *11* (Groth et al. 2013). In the BNA2 population, several QTLs were also identified on chromosomes *1*, *3*, *4*, *6*, *10* and *12* (Obidiegwu et al. 2015).

Wild *Solanum* species are a reservoir of resistance genes for many diseases, which is a valuable tool for breeders. Indeed, numerous resistance genes were discovered in wild *Solanum* species, such as *S. demissum* and *S. bulbocastanum* for late blight resistance or *S. tuberosum* ssp. *andigena* for resistance to viruses (Simko et al. 2007) and were introgressed in the potato breeding genepool. *Sen1* was probably present very early in the ancestors of cultivated potato as its frequency is high in potato breeding germplasm (Prodhomme et al. 2020a). Indeed, Khiutti et al. (2012) screened 52 landrace genotypes from *S. phureja*, *S. stenotomum*, *S. tuberosum* ssp. *andigenum* and *S. tuberosum* ssp. *tuberosum* with the *Sen1* linked marker Nl25 (Hehl et al. 1999), but they did not observe any correlation between wart resistance to pathotype 1 and taxonomy, ploidy level or geographic origin. This confirms our hypothesis that *Sen1* was already present in the ancestors of cultivated potato. Resistance to the higher pathotypes must, however, come from later introgressions, maybe as linkage drag during the introgression of *R* genes for other diseases such as nematodes or viruses. Several wild *Solanum* species have been reported to be potential sources for the higher pathotypes resistance. This is the case of *S. tuberosum* ssp. *andigena* (Bukasov and Kameraz 1959; Maris 1961; Ross 1986), *S. acaule* (Maris 1961; Ross 1986) or *S. demissum* (Bukasov and Kameraz, 1959; Maris 1961) which were reported several times as resistance sources. Recently, the *Sen3* gene could be traced back to the variety Ora, the pedigree of which contains *S. edinense* origins and a cultivar from Chiloe (Prodhomme et al. 2019). The *Sen2* gene was identified in a complex hybrid with *S. acaule*, *S. chacoense*, *S. demissum*, *S. gourlayi*, *S. microdontum*, *S. phureja*, *S. tuberosum*, *S. verrucosum* and *S. yungasense* in its pedigree (Plich et al. 2018). Knowing which species have been used to introgress resistance to wart disease in breeding material would be useful information for the elimination of redundancy in breeding germplasm and the identification of new resistances.

Therefore, we set out to identify potential additional *Sen* genes in potato breeding germplasm. First, we performed a Genome-Wide Association Study for resistance to pathotypes 2, 6 and 18 in the potato breeding genepool. The significantly associated markers were screened in three independent full-sib populations, resulting in the identification of *Sen4* on the south arm of chromosome *12* and *Rse*-*XIc*-*VTN62.33.3,* a QTL on the north arm of chromosome *11*, both bringing resistance to pathotypes 2, 6 and 18. Bulked segregant analyses (BSA) (Giovannoni et al. 1991; Michelmore et al. 1991) combined with Comparative Subsequence Sets Analyses (CoSSA) (Prodhomme et al. 2019) were used to find additional markers for *Sen4.* Also, CoSSA was pursued to map the resistance of Aventra, *Sen5,* on the south arm of chromosome *5*, involved in pathotypes 2, 6 and 18 resistance. Furthermore, CoSSA was used to design haplotype specific markers for the resistances segregating in the SaKa1 population (Ballvora et al. 2011). Finally, we identified a minor effect locus necessary to bring full resistance to pathotype 18 in combination with the *Sen3* gene (Prodhomme et al. 2019). Markers flanking *Sen1*, *Sen2*, *Sen3*, *Sen4*, *Sen5* and minor effect QTLs were screened in a panel of resistant and susceptible varieties and in a panel of wild *Solanum* accessions. The varieties and accessions sequenced in the study of Hardigan et al. (2017) were screened using CoSSA to identify the presence of *Sen1*, *Sen3*, *Sen4* and *Sen5*. The distribution over the breeding germplasm and the origin of the different *Sen* genes are inquired and discussed.

## Materials and methods

### Plant materials

For the GWAS, we used the genotypic dataset of 569 varieties produced with the 20 K SolSTW SNP array (Vos et al. 2015) and kept genotypes with a SNP call rate greater than 75%. We gathered phenotypic data for pathotypes 2, 6 and 18 (hereafter referred as P2, P6 and P18) from public and private sources (Supplementary File 1). The GWAS panels for P2, P6 and P18 were composed of 117, 138 and 53 genotypes, respectively (Supplementary File 1).

We used the five segregating populations AxV, AxD, KxA, SaKa1 and KxL to validate the GWAS results and to map novel and previously identified loci involved in potato wart disease resistance (Supplementary File 2). The AxV population was composed of 100 descendants of a cross between Axion (resistant to P1, P2, P6 and P18) and VR808 (resistant to P1). The AxD population resulted from a cross between Aventra (susceptible to P1, resistant to P2, P6 and P18) and Desiree (resistant to P1) and was composed of 42 descendants (Prodhomme et al. 2020a). The KxA population resulted from a cross between Kuras (resistant to P1) and Aventra and was composed of 35 descendants (Prodhomme et al. 2020a). KxA and AxD are half-sib populations, and to identify the resistance from Aventra, the two populations are together referred to as AxDK. The SaKa1 population (Ballvora et al. 2011) was composed of 124 descendants from the cross between Andante (resistant to P1, P2, P6 and P18) and Alegria (resistant to P1). The KxL population consisted of 328 descendants from a cross between Kuba (resistant to P1, P2, P6 and P18) and Ludmilla (resistant to P1) (Prodhomme et al. 2019).

To make an inventory of the potato wart disease resistance present in potato breeding germplasm and in wild *Solanum* species, we collected a panel of 118 potato breeding clones and varieties, called hereafter the variety panel. The variety panel was composed of resistant and susceptible varieties (mainly tetraploid), old and recent, bred in at least 11 different countries. In addition, a panel of 118 *Solanum* accessions, some from which wart resistance phenotypes were available through CGN (Centrum voor Genetische Bronnen Nederland), was compiled and called hereafter the Solanum panel. The Solanum panel was composed of diploid and polyploid accessions from the Solanum section petota and contained 38 different species. They originated from various regions ranging from Central and South America (Supplementary File 3).

### Phenotyping

The wart resistance phenotype data used in the GWAS were gathered from diverse sources such as National Lists, various websites, booklets from breeding companies and scientific papers (Supplementary File 1). The resistance scales used in the different sources were different, and for the purpose of GWAS, the quantitative scores were all transformed to a 1 (highly susceptible) to 10 (highly resistant) scale. Qualitative scores (i.e. resistant or susceptible) were transformed into quantitative scores, as indicated in Supplementary File 1. For each genotype, we calculated a final resistance score corrected for the source (origin of the phenotypic data) effect using restricted maximum likelihood (REML) as follows: ${\text{Resistance}} = {\text{source}} + {\text{genotype}}$, the source being included as a random effect and the genotype as a fixed effect.

The phenotypic assays performed on the mapping populations in this study are summarized in Supplementary Table 1. For the Spieckermann assays (Spieckermann and Kothoff 1924), each assessed tuber was given a quantitative score ranging from 10 (highly resistant, corresponding to the type 1 in Germany and the type “-” in the Netherlands) to 1 (highly susceptible, corresponding to the type 5 in Germany and to the type X in the Netherlands). A mean score was calculated for each genotype (Supplementary File 2). For the Glynne–Lemmerzahl assays (Glynne 1925; Lemmerzahl 1930), disease symptoms were rated from 1 (highly resistant, early defence necrosis) to 5 (highly susceptible) and mean scores were calculated between replicates. Pathotype 1 resistance scores for the populations AxD and KxA were previously described by Prodhomme et al. (2020a)). Phenotypic data for the KxL population were described by Prodhomme et al. (2019). Phenotypic data for the SaKa1 population were obtained by Ballvora et al. (2011).

For some *Solanum* accessions from the Solanum panel, phenotypic data for pathotypes 1, 2, 6 and/or 8 could be retrieved from the CGN (Centrum voor Genetische Bronnen Nederland) database (CGN 2019). Five to ten tubers per accession were phenotyped with the Glynne–Lemmerzahl method between 1980 and 1994. A qualitative score was given to the phenotyped accessions: resistant (R), intermediate (I) or susceptible (S) (Supplementary File 3).

### Genome-Wide Association Study of pathotypes 2, 6 and 18 resistance

The association analysis was carried out using two different models. The first model was a naive model which did not include a correction for the panel structure. For the second model, we calculated the van Raden kinship (VanRaden 2007) between the genotypes using a random subset of 1000 markers and performed a Principal Coordinate Analysis on the kinship. After comparing the inclusion of different numbers of principal coordinates (PCOs) as fixed effects in a mixed linear regression model (data not shown), we decided to include the first 30 PCOs to correct for the structure confounding effect. As potato resistance to the higher pathotypes is rare in potato germplasm, we reduced the minor allele frequency (MAF) threshold: for each panel, we kept markers present in at least three genotypes. Markers with more than 20% of missing data were removed from the dataset. In total, 12,279, 12,486 and 11,392 markers were used for the P2, P6 and P18 GWAS studies. The two GWAS models were fitted in GenStat version 18 (VSN International 2015). For each dataset (P2, P6, P18), the genome-wide significance threshold was calculated by the procedure QTHRESHOLD, using the method developed by Li and Ji (2005), similarly as described in Prodhomme et al. (2020a).

### Bulked Segregant Analysis (BSA) in the mapping populations

To design haplotype specific markers flanking *Sen4* segregating in AxV, a resistant bulk (AxV_RB) and a susceptible bulk (AxV_SB) were compiled containing 18 resistant and 15 susceptible descendants, respectively (Supplementary File 2). To identify the resistance locus from Aventra (resistant to P2, P6 and P18), we used the bulks previously compiled and sequenced by Prodhomme et al. (2020b). In the AxDK_Sen1_RB bulk, 15 out of 24 descendants showed strong resistance to other pathotypes than P1 (Supplementary File 2). The susceptible bulk AxDK_SB was composed of 10 descendants susceptible to all pathotypes. In the SaKa1 population, we selected 16 descendants resistant to P1, P2, P6 and P18 to build the SaKa1_RB bulk and 16 descendants resistant to P1 but susceptible to P2, P6 and P18 to build the SaKa1_P1RB bulk (Supplementary File 2). To identify minor effect loci providing full resistance to P18 in the KxL population from Prodhomme et al. (2019), we compiled one new bulk in this population. The KxL_P2P6RB bulk was composed of 17 descendants which were fully resistant to P2 and P6 but weakly susceptible to P18 with the Glynne–Lemmerzahl phenotyping method and contained the *Sen3* flanking markers.

### Comparative Subsequence Sets Analysis (CoSSA) workflows

Several CoSSA workflows have been conducted to identify the resistance loci segregating in the different populations. For each population, the workflow used was the CoSSA with reference genome described in Prodhomme et al. (2019). To summarize: R-bulk specific *k*-mers were selected by performing the difference between the R-bulk and the S-bulk. The R-bulk specific *k*-mers were filtered based on their frequency in function of the R-bulk sequencing depth: the *k*-mers selected had a frequency of $\frac{{R \text{-} bulk\,depth}}{4} \pm 0.5*\frac{{R \text{-} bulk\,depth}}{4}$. Because of the relatively low sequencing depth of the R-bulks from the AxV and Saka1 populations, we adapted the depth cut-off filter by increasing the lower and upper thresholds to improve the signal (Supplementary File 4). Next, the R-bulk specific *k*-mers were divided in function of their inheritance (from the R parent(s), referred to as resistance-specific *k*-mers; from the S parent(s); from all parents; from none of the parents). Where required, the difference was made between the resistance-specific *k*-mers and the *k*-mers present in susceptible varieties (called hereafter “minus S varieties”) to increase the haplotype specificity of the remaining *k*-mers. The resistance-specific *k*-mers (minus S varieties) were then mapped to the potato reference genome DM v4.03. The number of mapped *k*-mers per 1 Mb bin for each chromosome was counted and plotted. The details of each CoSSA workflow applied for each population are described in the Supplementary File 4.

CoSSA was also used to test the presence of *Sen1*, *Sen3*, *Sen4* and *Sen5* in the 67 varieties and accessions from Hardigan et al. (2017). The intersection between each of the above mentioned resistance gene specific *k*-mers (without the S varieties when applicable) was made with the *k*-mers from each of the 67 samples. The resulting *k*-mers were then mapped to the potato reference genome DM v4.03, *k*-mers per 1 Mb bin were counted, and the number of *k*-mers mapping to the *Sen1*, *Sen3*, *Sen4* and *Sen5* (fine)-mapped regions was compared with positive controls (varieties which hold the gene of interest).

### DNA extraction and sequencing

Genomic DNA of the parents of the populations (except SaKa1), of the bulked progeny clones and of susceptible varieties, was extracted from freshly harvested leaves according to Fulton et al. (1995) and purified using the Qiagen DNeasy Plant Mini Kit. The samples DNA concentration was measured using a Qubit Fluorometer (Invitrogen). For the different bulks, an equal amount of DNA from each progeny clone was pooled to obtain a final DNA quantity of 1 µg. For all the samples, 1 µg of (pooled) genomic DNA was used for the library preparation and sequenced on an Illumina platform. Paired-end (PE) reads of 151 bp were produced (Hartwig Medical foundation, Amsterdam, The Netherlands). A summary of the sequencing depth obtained for all sequenced samples is given in Supplementary File 4.

Lyophilized leaves from the SaKa1 population were obtained from SaKa Pflanzenzucht GmbH & Co. KG. The DNA of the genotypes in the bulks was extracted as described previously. The DNA concentration and quality were verified with a Qubit Fluorometer (Invitrogen) and on agarose gels. The DNA was partially degraded, probably due to storage of the lyophilized material at room temperature. Consequently, we compiled 4 sub-bulks in function of the level of degradation of the DNA as assessed on gel. The two resistant sub-bulks were named SaKa1_RB_small (containing samples with smaller DNA fragments), SaKa1_RB_big, and contained each eight individuals that were resistant to P1, P2, P6 and P18. SaKa1_P1RB_small and SaKa1_P1RB_big sub-bulks contained both eight individuals that were resistant to P1 and were positive for the *Sen1* markers, but that were susceptible to P2, P6 and P18. During sequencing library preparation, the two sub-bulks with smaller DNA fragments were less sheared than the two with big DNA fragments. The four sub-bulks were sequenced as described above. Appropriate sequencing quality and yield of the four sub-bulks was confirmed using FastQC (Andrew 2010) and the small and big fragments sub-bulks were merged in silico to form SaKa1_RB and SaKa1_P1RB bulks for subsequent CoSSA.

Genomic DNA of the different siblings, parents and the variety panel was extracted from freshly harvested leaves or from tubers according to Fulton et al. (1995). The DNA quality was assessed on agarose gels. Genomic DNA from the Solanum panel was extracted by the Dr Van Haeringen Laboratorium (Wageningen, The Netherlands). The DNA concentration was measured using a NanoDrop ND-1000 spectrophotometer (Thermo Scientific).

### KASP and PCR markers

Kompetitive Allele Specific PCR (KASP) markers were designed as described in Prodhomme et al. (2019). Adjusted concentrations of 5–50 ng/μl of genomic DNA were used for the KASP assays. The KASP assays were performed in house or by C. Meijer BV (Rilland, The Netherlands) as described in Prodhomme et al. (2019). PCR marker 5450_3, as described by Plich et al. (2018), was used to test the presence of *Sen2* in the variety and Solanum panels. Concentrations of 5–10 ng/µl of genomic DNA were used to amplify a 1046 bp fragment. *Tas*I restriction enzyme fragments were visualised on 2% agarose gels. Genomic DNA from DG 97–264 (Plich et al. 2018) was used as a positive control, and its 1046 bp amplicon was digested into fragments of eight different sizes.

We used Chi^2^ test to verify the goodness of fit of the segregation ratio of the tested KASP markers in the five populations with the expected ratio (defined in function of the dosage observed in the parents). We used Kruskal–Wallis test to validate the association of the tested markers with resistance. All the statistical tests were performed on R v3.2.3.

### Phylogenetic analysis in the Solanum panel

Genotypic data from 222 AFLP markers were available for 108 of the 118 accessions from the Solanum panel (Jacobs et al. 2008). We used Mesquite v3.6 (Madison and Madison 2018) to format the data and MrBayes v3.2.7 (Huelsenbeck and Ronquist 2001) to infer a Bayesian rooted tree. MrBayes was run with four chains, 10,000,000 generations, a sampling frequency of 10,000 and a temperature setting for the heated chains of 0.25. Six *S. etuberosum* accessions were added as an outgroup to the analysis in order to root the phylogenetic tree.

## Results

### GWAS of pathotypes 2, 6 and 18 resistance identifies a minor and a major effect QTL

**Fig. 1:**
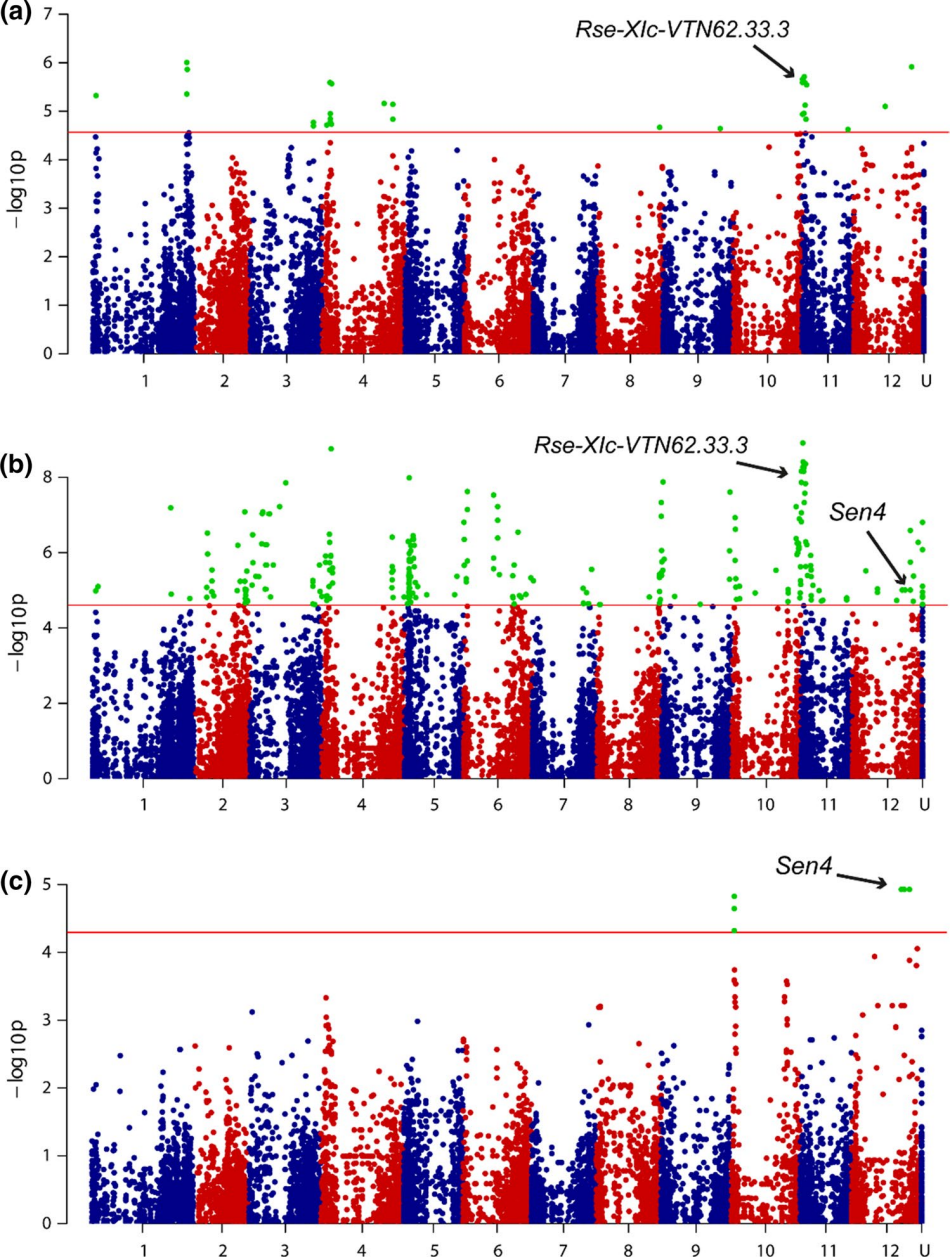
GWAS performed on pathotypes 2, 6 and 18 resistance. Manhattan plots of the GWAS performed on **a** P2, **b** P6 and **c** P18 resistance. The x axis represents the 12 potato chromosomes. Markers from unanchored scaffolds (also referred to as Chr0), chloroplast and mitochondrion markers are indicated by U. The horizontal red line is the threshold of significance as calculated by the method of Li and Ji (2005). Significant markers above the threshold are highlighted in green. The markers from the *Sen4* haplotype and from the chromosome *11* haplotype which is associated with resistance in the AxD, KxA and AxV populations are indicated. The SNP array was designed with an emphasis on 800 genes on gene rich arms and avoiding pericentromeric heterochromatin, which resulted in artificial peaks towards the end of the chromosomes (color figure online)

In order to identify SNPs and loci involved in potato wart disease resistance, we performed a GWAS study in a panel of varieties that was previously described and genotyped (Vos et al. 2015). With the naive GWAS model, 32, 292 and 9 markers were significantly associated with P2, P6 and P18, respectively (Fig. 1). The correlation between the first 30 PCos of the PCoA and P2, P6 and P18 resistance was of 0.72, 0.70 and 0.79, respectively. Remarkably, with the PCoA corrected model, none of the markers reached the significance thresholds of 4.567, 4.608 and 4.295 for P2, P6 and P18, respectively, suggesting that resistance is present in related genotypes. Pursuing with the results of the naive model, we found that 23 SNPs were significantly associated with both P2 and P6, ten of which were located on the north arm of chromosome *11* between 0.81 and 4.35 Mb. Seven SNPs were associated with both P6 and P18 resistance, six of which were located on the south arm of chromosome *12* between 43.3 and 50.4 Mb. We set out to validate a subset of the identified SNPs through their linkage with wart disease resistance that is segregating in the sibling populations (AxV, AxD, KxA; Phenotyping data from these populations can be found in Supplementary Figures 1 to 3 and Supplementary File 2). From the total number of 303 GWAS SNPs, we selected 42 SNPs having a positive effect on resistance and having the minor alleles present in simplex or duplex in the resistant parents Axion and Aventra and absent from the susceptible parents. These 42 selected SNPs were converted to KASP markers in order to test their segregation in the three sibling populations (Genotyping and phenotyping data from these populations have been collected in Supplementary File 2).

Three markers from chromosome *12* located between 43.3 and 50.4 Mb (PotVar0031912, PotVar0036325 and PotVar0037666) co-segregated and were strongly associated with P2, P6 and P18 resistance in the AxV population (Supplementary File 5). This chromosome *12* haplotype showed a strong effect on resistance (Supplementary Figure 4). Therefore, we concluded that this was a major effect QTL and gave it the name of *Sen4*, following the naming system of the dominant, major effect *S. endobioticum* resistance genes. The oldest variety in which we found the *Sen4* markers in the GWAS panels is Alcmaria (Supplementary File 1).

Another twelve markers from the north arm of chromosome *11* were significantly associated with resistance in AxV, albeit with a lower effect on resistance than *Sen4.* The effect of *Sen4* was stronger for P6 (mean scores of 9.5 and 8.5 for *Sen4* and the QTL on chromosome *11*, respectively) and especially for P18 resistance (mean scores of 9.1 and 6.0; Supplementary Figure 4, Supplementary File 5). We therefore consider this a minor effect QTL and called this haplotype *Rse*-*XIc*-*VTN62.33.3*. With respect to the naming of major and minor effect QTLs, we followed the naming system from Obidiegwu et al. (2014) and added the name of the oldest clone in which the haplotype markers were found in order to distinguish different haplotypes with overlapping genomic locations. An overview of the different QTLs that we found in this study is provided in Table 1. The effect of *Sen4* was stronger than the effect of *Rse*-*XIc*-*VTN62.33.3* for P6 (mean scores of 9.5 and 8.5 for *Sen4* and *Rse*-*XIc*-*VTN62.33.3*, respectively) and especially for P18 resistance (mean scores of 9.1 and 6.0; Supplementary Figure 4). The highest level of resistance was achieved when both loci were present.

**Table 1:** Nomenclature and description of major and minor resistance QTLs

Resistance QTLs		Type of QTL	Chr.	Position (Mb)^b^	Resistance spectrum	R parent in this study
This study	Previous study^a^					
*Rse*-*Ib*-*Andante*-*a*	*Rse*-*Ib*-*a* Ballvora et al. (2011)	Minor	1	~ 72–8	P1, P2, P6, P18	Andante
*Rse*-*Ib*-*Andante*-*c*		Minor	1	~ 70–76	P1, P2, P6, P18	Andante
*Sen5*		Major	5	[49.2–51.16]	P2, P6, P18	Aventra
*Rse*-*VIIIb*-*Kuba*		Minor	8	~ 44–47	P18	Kuba
	*Rse*-*VIIIa* Groth et al. (2013)	Minor	8	South arm chr. 8	P1, P2, P6, P18	Saturna, Panda
*Rse*-*IXa*-*Andante*	*Rse*-*IXa*-*a* Ballvora et al. (2011)	Minor	9	~ 55–61.5	P18	Andante
*Rse*-*IXb*-*Ludmilla*		Minor	9	~ 55–57	P2, P6, P18	Ludmilla
*Sen1*	*Sen1* Hehl et al. (1999)	Major	11	[1.31–1.67]	P1	Desiree, Kuras
*Sen3*	*Rse*-*XIa* Bartkiewicz et al. (2018)	Major	11	[1.26–1.52]	P1, P2, P6, P8?, P18	Kuba
*Rse*-*XIc*-*VTN62.33.3*		Minor	11	~ 0–5	P2, P6, P18	Axion, Aventra
	*Sen2* Plich et al. (2018)	Major	11	[33.7–35.06]	P1, P2, P3, P6, P8, P18, P39	DG 97-264
*Rse*-*XId*-*Andante*		Minor	11	~ 0–31	P2, P6, P18	Andante
*Sen4*		Major	12	[48.5–51.5]	P2, P6, P18	Axion
	*Rse*-*XIIa* Obidiegwu et al. (2015)	Minor	12	fUll chr 12	P2, P6, P18	Karolin, Ps-354

^a^The “Rse-chromosome number” naming is according to Obidiegwu et al. (2015). The name of the oldest donor was added. At the end of the locus name, if needed, a letter was added to differentiate the different haplotypes or allelic variants

^b^[–]: interval of the closest flanking markers, ~: peak interval in CoSSA results

### CoSSA to fine-map *Sen4*

**Fig. 2:**
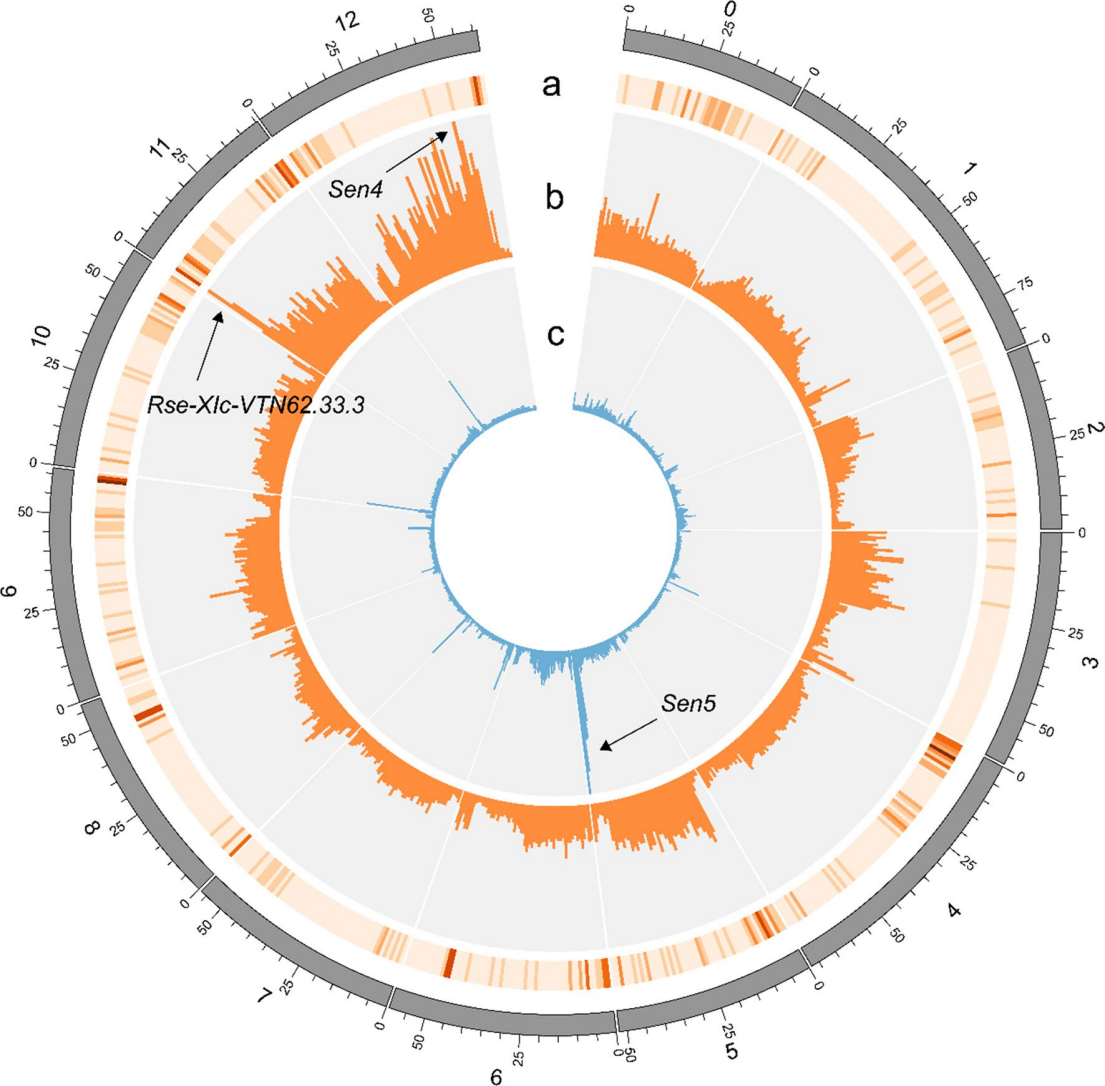
Comparative Subsequence Sets Analysis to fine-map *Sen4* and identify *Sen5*. **a** Number of NLR genes per bin of 1 Mb from the potato reference genome DM v4.03 according to Jupe et al. (2013)). **b** CoSSA performed in the AxV population. 91,868,050 unique (Nu) AxV_RB specific k-mers were identified from a total number (Nt) of 492,018,414 k-mers that occurred at a frequency from 4 to 20 (Supplementary File 4). From these k-mers, 42% (Nu = 38,573,330; Nt = 195,954,466) were inherited from Axion. From these Axion resistance-specific k-mers, we removed k-mers from the susceptible varieties Alegria, Desiree, Kuras and Ludmilla to select for haplotype specific k-mers. This last set was composed of 17,440,189 unique and 88,818,285 total k-mers. These AxV_RB specific *k*-mers inherited from Axion minus the S varieties *k*-mers were mapped to the reference genome (*y*_max_ = 67,500 *k*-*mers*). **c** CoSSA performed in the AxDK population. 32,183,695 unique k-mers (Nt = 267,071,227) specific to the AxDK_Sen1_RB bulk (Supplementary File 4). From these k-mers, 18% (Nu = 5,934,080, Nt = 48,978,404) were inherited from the resistant parent Aventra. From these Aventra resistance-specific k-mers, commonly occurring k-mers were removed by subtracting k-mers from the susceptible varieties Alegria, Ludmilla and VR808, resulting in a subset of 3,533,028 unique k-mers (Nt = 28,794,943). The AxDK_RB specific *k*-mers inherited from Aventra minus the S varieties *k*-mers were mapped to the reference genome (*y*_max_ = 58,000 *k*-mers)

**Fig. 3:**
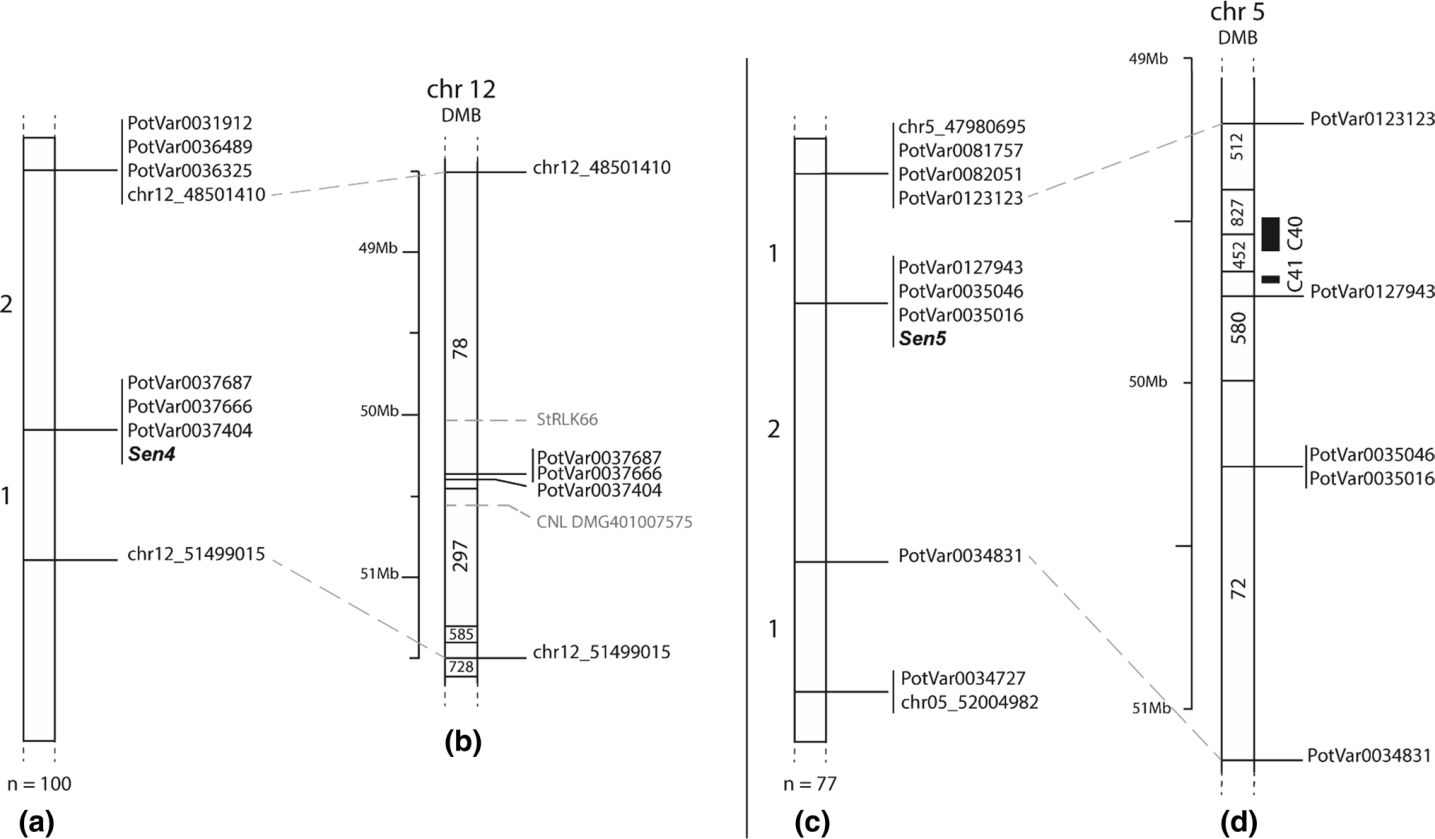
Genetic and physical maps of *Sen4* and *Sen5.* Genetic maps of *Sen4* (**a**) and *Sen5* (**c**) in the AxV and AxDK populations, respectively. The number of recombinants found between the screened markers is given on the left side of the maps. Physical maps of *Sen4* (**b**) and *Sen5* (**d**) according to the potato reference genome DM v4.03. On the right side of the physical maps are shown the screened markers, the RLK genes (grey font) according to Nazarian-Firouzabadi et al. (2019), the NLR genes (grey font) and clusters (black boxes) according to Jupe et al. (2013)

To further characterize the resistance from Axion, we pursued a CoSSA approach. We compiled and sequenced a resistant bulk (AxV_RB) from 18 selected AxV descendants which were strongly resistant to P6 and P18, and a susceptible bulk (AxV_SB) composed of 15 descendants that were highly susceptible to P6 and P18 (Supplementary File 2). As shown in Fig. 2, two main peaks could be identified on chromosomes *11* and *12*. Firstly, a peak composed of 163,965 Axion resistance-specific *k*-mers was observed on the north arm of chromosome *11* between 1 and 3 Mb. The chromosome *11* peak included 4 of the 12 SNPs that were validated in AxV (PotVar0066337, PotVar0067017, PotVar0106057 and PotVar0106019) and that defined *Rse*-*XIc*-*VTN62.33.3*. This re-identification of *Rse*-*XIc*-*VTN62.33.3* confirmed the contribution of this QTL to resistance. Secondly, from these Axion resistance-specific *k*-mers, 10% (Nu = 1,752,553) mapped to a broad peak ranging from 10 Mb until 54 Mb on chromosome *12* (Fig. 2) and included the three markers (PotVar0031912, PotVar0036325 and PotVar0037666) that defined *Sen4* in the GWAS. To design markers flanking *Sen4*, we sought additional SNPs in the chromosome *12* peak. We selected three SNPs to which *Sen4* specific *k*-mers were matching and for which PotVar markers had already been designed (Uitdewilligen et al. 2013; PotVar0036489, PotVar0037687 and PotVar0037404). Another two SNPs (chr12_ 48501410 and chr12_51499015) were selected solely on the CoSSA data and were used to design novel KASP markers. Among 100 AxV descendants, only three recombinants (AxV_13_33, AxV_13_84 and AxV_13_38; Supplementary File 2) were found between the outermost selected SNPs. Unfortunately, recombinant AxV_13_38 also held *Rse*-*XIc*-*VTN62.33.3* and was therefore not informative for *Sen4* mapping. Consequently, we could map *Sen4* in a 3 Mb region between 48.5 and 51.5 Mb (between chr12_ 48501410 and chr12_51499015) (Fig. 3a, b).

### CoSSA to identify *Sen5*

In our second and third validation populations (AxD and KxA), which are half sib populations of Aventra, we also tested the *Rse*-*XIc*-*VTN62.33.3* KASP markers. *Rse*-*XIc*-*VTN62.33.3* was significantly associated with P6 and P18 as assessed by the Glynne–Lemmerzahl method and P18 as assessed by the Spieckermann method in AxD (Supplementary File 5). In KxA, *Rse*-*XIc*-*VTN62.33.3* was significantly associated with P2 and P6 in the Glynne–Lemmerzahl method and P18 in the Spieckermann method. None of the *Rse*-*XIc*-*VTN62.33.3* markers were associated with P1 resistance, ruling out the possibility that *Rse*-*XIc*-*VTN62.33.3* and *Sen1* are present in the same haplotype.

Several descendants in the AxD and KxA populations which did not hold *Rse*-*XIc*-*VTN62.33.3* showed a strong resistance to P2, P6 and P18, suggesting that at least one other resistance locus was segregating. To identify the additional resistance(s) segregating in AxD and KxA, we re-used the bulks that were used to fine-map *Sen1* (AxDK_Sen1_SB and AxDK_Sen1_RB) (Prodhomme et al. 2020b). All 10 individuals from the susceptible bulk (AxDK_Sen1_SB) were highly susceptible to all the tested pathotypes. Within AxDK_Sen1_RB, 15 out of 24 individuals showed strong resistance to P2, P6 and/or P18 while the other nine were only resistant to P1. To identify *k*-mers linked to P2, P6 and P18 resistance, we adapted the *k*-mers frequency threshold and retained *k*-mers with a depth between 6x and 17x in BR (The resistant haplotype(s) from Aventra were expected to have a sequencing depth between 6x and 17x as AxDK_Sen1_RB had a sequencing depth of ~ 72x, 15 out of 24 descendants were resistant, and assuming that a single simplex locus was causal for resistance). CoSSA resulted in 207,335 Aventra resistance-specific *k*-mers that mapped to the south arm of chromosome *5* and produced a peak between 47 and 52 Mb (Fig. 2). To validate the association of the chromosome *5* locus with resistance, we identified eight PotVar SNPs from Uitdewilligen et al. (2013) that matched Aventra resistance-specific *k*-mers (as described in Fig. 2). We also designed two new markers flanking the *k*-mers peak. All ten markers co-segregated (Fig. 3c) and were strongly associated with P2, P6 and P18 in AxD and in KxA (Supplementary File 5). The effect of the chromosome *5* haplotype on resistance was stronger than the effect of *Rse*-*XIc*-*VTN62.33.3* in AxD for P2 (mean resistance scores of 1.9 and 2.8 for the chromosome *5* locus and *Rse*-*XIc*-*VTN62.33.3*, respectively) with Glynne–Lemmerzahl and for P18 with Spieckermann (mean resistance scores of 8.0 and 5.6, respectively; Supplementary Figure 5). In KxA, the chromosome *5* haplotype provided a stronger effect on resistance for P6 (mean scores of 3.5 and 4.0 for the chromosome *5* locus and *Rse*-*XIc*-*VTN62.33.3*, respectively) and P18 (mean scores of 3.7 and 4.1, respectively) with Glynne–Lemmerzahl and P18 (mean scores of 8.1 and 4.9, respectively) with Spieckermann (Supplementary Figure 6). Again, the highest level of resistance was achieved when both *Sen5* and *Rse*-*XIc*-*VTN62.33.3* were present. Because Aventra chromosome *5* resistance is a strong effect locus, we decided to name it *Sen5*.

To further delimit the *Sen5* genetic interval, we studied recombinants. The two recombinants from AxD are not informative as they hold *Rse*-*XIc*-*VTN62.33.3*. The two KxA recombinants do not hold *Rse*-*XIc*-*VTN62.33.3* and were clearly susceptible to P6 and P18 in the Spieckermann assay. Therefore, we could map *Sen5* to a 1.96 Mb region of the south arm of chromosome *5* between 49.2 and 51.16 Mb (between PotVar0123123 and PotVar0034831) (Fig. 3d).

### CoSSA in the SaKa1 population

In the SaKa1 population, resistance to P1 was skewed towards resistance, whereas resistance to P2, P6 and P18 was skewed towards susceptibility (Ballvora et al. 2011). The segregation of P1 resistance was bimodal, whereas resistance to the other pathotypes was quantitative. A PCA performed on the phenotypes showed that the descendants could be divided in three different groups: group 1 contained 37 descendants which were resistant to the four pathotypes, group 2 contained 47 descendants which were only resistant to P1 and group 3 contained 39 descendants susceptible to the four pathotypes (Supplementary Figure 7). To identify the loci providing resistance to P2, P6 and P18, we compiled the SaKa1_RB bulk composed of 16 descendants from group 1 (resistant to the four pathotypes) and the SaKa1_P1RB bulk composed of 16 descendants from group 2 (only resistant to P1) (Supplementary File 2). A peak of 90,013 *k*-mers inherited from Andante was found on the south arm of chromosome *1* between 72 and 80 Mb (Supplementary Figure 8A). A second peak of 62,861 *k*-mers inherited from Andante was found on the south arm of chromosome *9* between 55 and 61.5 Mb. A third broad peak on chromosome *11* between 0 and 31 Mb was composed of 152,829 *k*-mers from Andante. A fourth peak of 54,300 *k*-mers inherited from Alegria was observed on the south arm of chromosome *12* between 57 and 60 Mb (this latter interval was not overlapping with the *Sen4* locus). KASP markers were designed to validate the association of these peaks with resistance. The *Sen1* flanking markers (Prodhomme et al. 2020b) were also included in this screening.

The *Sen1* flanking markers were significantly associated with P1 resistance in the SaKa1 population (Supplementary File 5). The effect of *Sen1* on P1 resistance was very strong, with a mean resistance score of 2.19 when *Sen1* was present and 3.56 when *Sen1* was absent (Supplementary Figure 9). The markers on the south arm of chromosome *1* between 72.77 and 79.69 Mb were also associated with P1 resistance albeit with a lower effect than the *Sen1* markers (mean resistance score of 2.4 for *Sen1* alone, mean score of 3.45 for chr01_76425362 alone). The resistance to P1 was greatly improved when both loci were present (mean score of 2.13; Supplementary Figure 9).

This chromosome *1* locus, which we called *Rse*-*Ib*-*Andante* (Table 1), was also associated with P2, P6 and P18 resistance (Supplementary File 5). The marker with the strongest effect on resistance was chr01_76425362 which segregated in a 1:4:1 (nulliplex:simplex:duplex) ratio. The resistance was stronger when the marker was present in duplex (Supplementary Figure 9) which can be due either to a dosage effect or to the fact that chr01_76425362 is present on two different haplotypes that both bring resistance. The markers chr01_72774086, chr01_74148509, chr01_74162620, chr01_77750280, chr01_77801278, chr01_79026840 and chr01_79694600 were all linked to each other and one copy of chr01_76425362 showing that they were located on the same haplotype of chromosome *1* (referred hereafter as *Rse*-*Ib*-*Andante-*a).

Markers located under the 0–31 Mb peak of chromosome *11* were linked to each other and were also significantly associated with P2, P6 and P18 resistance. The effect of this locus, that we called *Rse*-*XId*-*Andante,* on resistance was weaker than *Rse*-*Ib*-*Andante*-*a*, but the resistance was improved when both loci were present (Supplementary Figure 9). The markers designed under the chromosome *9* peak were also linked to each other and significantly associated with P18 resistance only. The effect of this locus, that we called *Rse*-*IXa*-*Andante*, on P18 resistance, was weaker than when chr01_76425362 was present in duplex but stronger than *Rse*-*XId*-*Andante*. The concomitant presence of chr01_76425362, *Rse*-*IXa*-*Andante* and *Rse*-*XId*-*Andante* brought a strong resistance to P18 (mean score of 2.37; Supplementary Figure 9).

*Rse*-*Ib*-*Andante*-*a* was associated with P1, P2, P6 and P18 resistance but not all the descendants which hold this haplotype were resistant to P2, P6 and P18. This observation suggests that *Rse*-*Ib*-*Andante*-*a* is dependent on other QTLs in the background. In an attempt to identify the other locus/loci required for *Rse*-*Ib*-*Andante*-*a* to bring full resistance, we compiled two new bulks, SaKa1_chr01_RB and SaKa1_chr01_SB, composed, respectively, of 17 descendants holding *Rse*-*Ib*-*Andante*-*a* and fully resistant to the four pathotypes and of 17 descendants holding *Rse*-*Ib*-*Andante*-*a* but with a weak resistance to the four pathotypes (Supplementary File 2). In a new CoSSA, the difference between the *k*-mers from SaKa1_chr01_RB and SaKa1_chr01_SB was made (Supplementary File 4). A high peak composed of 113,781 SaKa1_chr01_RB bulk specific *k*-mers inherited from Andante was observed on chromosome *1* between 67 and 74 Mb, overlapping with the position of *Rse*-*Ib*-*Andante*-*a* (Supplementary Figure 8B). Two markers were designed under this peak (chr01_70066624 and chr01_73527005). Both markers were present on the same haplotype (called *Rse*-*Ib*-*Andante*-*c*) which did not co-segregate with *Rse*-*Ib*-*Andante*-*a*. *Rse*-*Ib*-*Andante*-*c* was significantly associated with P2, P6 and P18 resistance (Supplementary File 5). This was in agreement with our hypothesis that marker chr01_76425362 was present on two different haplotypes (or alleles) from *Rse*-*Ib*-*Andante* that both contribute to resistance. When comparing the presence/absence of *Rse*-*Ib*-*Andante*-*a*and *Rse*-*Ib*-*Andante*-*c* markers, it appeared that marker chr01_76425362 was indeed present on both haplotypes. In another CoSSA analysis, the difference between SaKa1_chr01_SB and SaKa1_chr01_RB was made to identify haplotypes which potentially contributed to susceptibility (Supplementary File 4). A high peak of 215,605 SaKa1_chr01_SB bulk specific *k*-mers inherited from Andante was observed again on the south arm of chromosome *1* between 74 and 84 Mb. Two markers were designed under this peak (chr01_77329972 and chr01_79751572). Both were located on the same haplotype (*Rse*-*Ib*-*Andante*-*b*) and showed a significant association with P1, P2 and P6 susceptibility. This is most likely due to a repulsion effect of *Rse*-*Ib*-*Andante*-*b* with the haplotypes *Rse*-*Ib*-*Andante*-*a* and *Rse*-*Ib*-*Andante*-*c*. These results reveal a complex architecture of wart disease resistance in the SaKa1 population which involves several haplotypes of the *Rse*-*Ib*-*Andante* locus. Despite the fact that *Rse*-*Ib*-*Andante*-*a* had a major effect on resistance, we decided to include it in the *Rse* naming system for minor effect wart resistance QTLs because of its co-dominance with *Rse*-*Ib*-*Andante*-*c.*.

### CoSSA to identify minor effect loci in the KxL population

In the KxL population (*n* = 328) from Prodhomme et al. (2019), the major effect resistance gene *Sen3* was segregating. In addition, minor QTLs that provided full P18 resistance to Kuba seemed to be segregating as well. This became clear when we performed a principal component analysis (PCA) on the P2, P6 and P18 resistance scores assessed with the Glynne–Lemmerzahl method (Supplementary Figure 10), and three groups could be distinguished. Group 1 was composed of 67 descendants that were fully resistant to P2, P6 and P18. Group 2 contained 92 descendants resistant to P2 and P6 but slightly susceptible or weakly resistant to P18. Group 3 contained 169 descendants susceptible to P2, P6 and P18. The proportion of group 1 + 2 and 3 reflected a 1:1 segregation ratio and *Sen3* co-segregated with group 1 and 2 phenotypes (except for 5 false positives out of 328). The groups 1 and 2 represent each 25% of the KxL population, suggesting that there is another locus segregating in a 1:1 ratio which is required by *Sen3* to bring full resistance to P18. To identify this locus, we compiled the KxL_P2P6RB bulk which was composed of 17 individuals belonging to phenotypic group 2. We re-used the KxL_RB bulk from Prodhomme et al. (2019) which consisted of 17 individuals of group 1. CoSSA identified four peaks of KxL_RB bulk specific *k*-mers that were inherited from Kuba, and six peaks that were inherited from the susceptible parent Ludmilla (Supplementary Figure 11). We designed KASP markers to validate eight of these peaks and screened the entire offspring (Supplementary File 5). Only the two markers located on the south arm of chromosome *8* were significantly associated with P18 resistance obtained with the Glynne–Lemmerzahl method. This chromosome *8* locus, that we called *Rse*-*VIIIb*-*Kuba*, significantly improved P18 resistance in combination with *Sen3*. Although the *Rse*-*VIIIb*-*Kuba* effect alone was very small compared to *Sen3* alone (Supplementary Figure 12).

### Inventory of the wart disease resistances present in the potato breeding gene pool

A panel of 118 potato varieties and clones was screened with the flanking markers of major *Sen* genes and minor effect QTLs as identified in this study or as known from literature. Unfortunately, several of these minor effect QTLs KASP markers did not perform well and data could only be obtained for *Rse*-*Ib*-*Andante*-*a*, *Rse*-*Ib*-*Andante*-*ac* (chr01_76425362), *Rse*-*VIIIb*-*Kuba*, *Rse*-*IXb*-*Ludmilla* (chr09_55113777; Prodhomme et al. 2019), *Rse*-*XIc*-*VTN62.33.3*, and *Rse*-*XId*-*Andante* (Supplementary File 3). Besides KASP markers analysis on the 118 potato varieties, 23 varieties whose genomes were sequenced by Hardigan et al. (2017) were screened for the *Sen1*, *Sen3*, *Sen4* and *Sen5* genes using CoSSA (Table 2; Supplementary File 6). *Sen1* was present in 59.6% (*n* = 84) of the tested varieties, 78 of which are resistant to P1, two have an intermediate level of resistance and for four the resistance is unknown. *Sen1* is not present in any of the varieties susceptible to P1. *Sen2* marker was absent from all the varieties except for the variety Bonza which showed a banding pattern which suggests it might be positive for *Sen2*. *Sen3* was found in 22 varieties, 17 of which are known to be resistant to P1 and at least one of the higher pathotypes. Thirteen of the *Sen3* varieties hold BRA9089 in their pedigree, for the rest, the pedigree is incomplete or unknown. *Sen4* was found in 13 varieties, ten of which are resistant to P2, twelve to P6, and nine to P18. Only Berber is susceptible to P2, P6 and P18. The oldest clone in which we identified *Sen4* is AM78-3704 and nine of the *Sen4* varieties hold AM78-3704 in their pedigree. In the GWAS genotypic data, the *Sen4* markers were found in Alcmaria, the grand-parent of AM78-3704 (Supplementary File 1). Among the twelve resistant varieties holding *Sen4* in our variety panel, ten hold Alcmaria in their pedigree, making it a very likely source of resistance. In the variety panel, only Aventra, Kanjer and the breeding clone VE71-105 hold *Sen5.* Aventra is resistant to the three higher pathotypes, and Kanjer is resistant to P2 and P6 (it is slightly susceptible to P1 and its resistance to P18 is unknown). VE71-105 has an intermediate level of resistance for P2, is resistant to P6 and susceptible to P18. Aventra’s pedigree is unknown, and Kanjer holds VE71-105 in its pedigree (van Berloo et al. 2007).

**Table 2:**
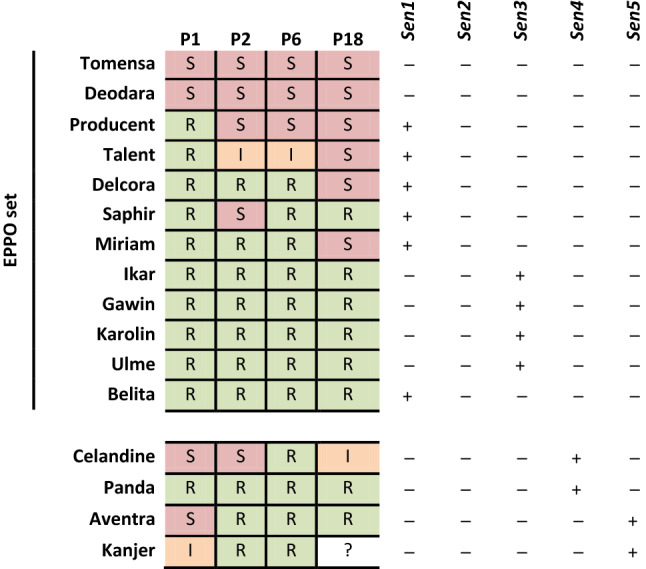
*Sen* genes resistance spectrum

*Rse*-*Ib*-*Andante*-*a* was found in 22 varieties of the panel, including five genotypes which are known to be susceptible to P2, P6 and P18. Marker chr01_76425362, present on haplotypes a and c of this locus, was present in 47 varieties. Again, there was no clear correlation with resistance to the higher pathotypes.

*Rse*-*VIIIb*-*Kuba* was identified in ten varieties from the panel. Three of them are known to be resistant to P18 (Actaro, Kuba and Smart), but three of them are known to be susceptible (Delcora, Talent, VR808). This is in agreement with our study of the KxL population where we found that *Rse*-*VIIIb*-*Kuba* alone did not show a pronounced resistance effect, while combined with *Sen3* it enhances the resistance level.

*Rse*-*IXb*-*Ludmilla* was found in twelve varieties, including susceptible ones, which was expected, as also in Ludmilla it did not provide resistance on its own. Finally, *Rse*-*XIc*-*VTN62.33.3* and *Rse*-*XId*-*Andante* were identified in 27 and 30 varieties, 21 and 17 of which, respectively, show resistance to at least one of the higher pathotypes.

### *Sen* resistance gene recognition spectrum and representation in the EPPO panel

After identifying multiple major and minor effect QTLs, it remains important to identify the pathogen recognition spectrum. One way to pursue this question is to identify genotypes which hold only one of the QTLs. Among the 56 varieties which hold *Sen1* but do not hold any of the other identified major *Sen* genes, 53 are resistant to P1 and 20 do not show resistance to any of the higher pathotypes (Supplementary File 3). The resistance spectrum of *Sen1* is therefore specific to pathotype 1 of *S. endobioticum*, as observed previously in several studies (Ballvora et al. 2011; Gebhardt et al. 2006; Groth et al. 2013; Plich et al. 2018). *Sen2* was shown to bring resistance to P1, P2, P3, P6, P8, P18 and P38 (Plich et al. 2018). In our variety panel, the *Sen2* marker was observed in Bonza which is confirmed to be resistant to pathotypes 1, 2 and 6. In our variety panel, there are 11 varieties which hold *Sen3* without *Sen1*, ten of which are known to be resistant to P1 (Table 2; Supplementary File 3). Moreover, five of these varieties are also resistant to pathotype 8. These observations suggest that *Sen3* is involved in resistance to pathotypes 1, 2, 6, 8 and 18. *Sen4* was shown to bring resistance to pathotypes 2, 6 and 18 in the AxV population. In the variety panel, only two varieties hold *Sen4* without *Sen1,* one being susceptible to P1 (Celandine) and one resistant (Panda) (Table 2). Therefore, it is hard to make unambiguous conclusions about an extended resistance spectrum of *Sen4. Sen5* is present in two varieties that do not hold *Sen1* and are susceptible (Aventra) or slightly susceptible (Kanjer) to P1 (Table 2). This observation and the study we made in the AxD and KxA populations show that the resistance spectrum of *Sen5* is specific to pathotypes 2, 6 and 18.

Among the varieties from the EPPO set (EPPO 2004, 2017), *Sen1* was found in six of the ten varieties resistant to P1 (Table 2). *Sen3* was present in the four remaining varieties resistant to P1 which are also resistant to P2, P6 and P18. Neither *Sen2*, *Sen4 n*or *Sen5* was found in the EPPO set varieties. The resistance present in Talent, Delcora, Saphir, Miriam and Belita is not explained by any of the *Sen* genes identified so far.

### Resistance in *Solanum* species

**Fig. 4:**
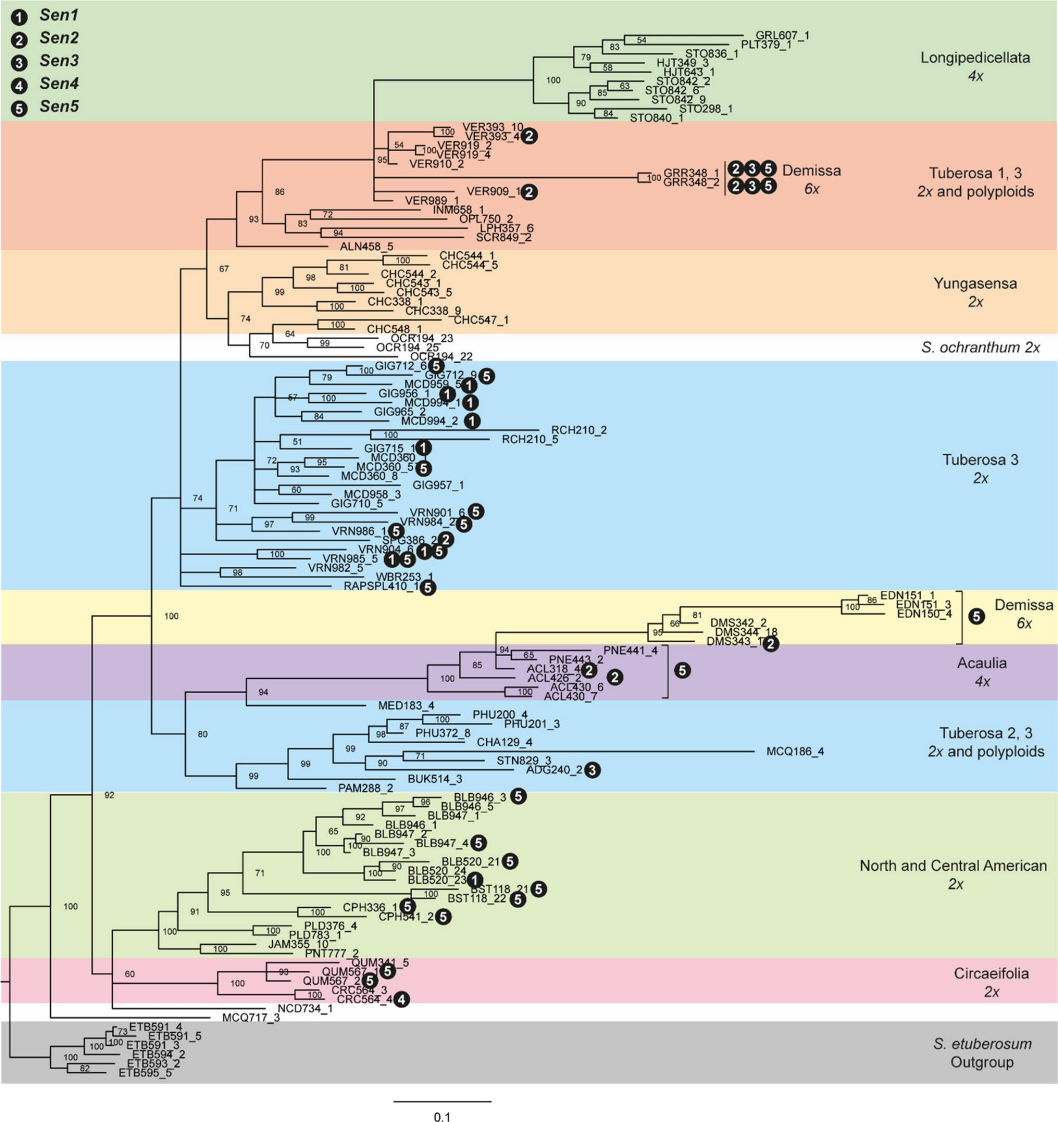
Classification of *Solanum* accessions screened for the *Sen* genes. Bayesian rooted tree of 108 of the 118 *Solanum* accessions screened for the *Sen* genes, based on the genotypic data of 222 AFLPs that were generated in a previous study (Jacobs et al. 2008). Branch lengths are proportional to the number of changes/site, and posterior probabilities are shown at each node. The classification is adapted from Hawkes’ series (Hawkes 1990). The *S. etuberosum* accessions were included in the phylogenetic analysis to form the outgroup, but they were not screened for the *Sen* genes. The presence of *Sen1*, *Sen2*, *Sen3*, *Sen4* and *Sen5* is based on markers chr11_1666351, Sen2_CAPS, chr11_1772869, PotVar0037404 and PotVar0034831, respectively

Wart disease resistance breeding has introduced resistances from crop wild relatives which resulted from co-evolution between host and pathogen. In an attempt to trace the donor species of the resistances under study, we screened a panel of 118 accessions from 38 wild *Solanum* species (Fig. 4) with markers flanking the *Sen* genes and the minor effect QTLs (Supplementary File 3). An additional set of 44 wild species and landraces (Hardigan et al. 2017) was analysed using CoSSA for the presence of *Sen1*, *Sen3*, *Sen4* and *Sen5*. The *Sen1* linked marker chr11_1666351 was found in seven accessions of the Tuberosa 3 series (Hawkes 1990) and one diploid *S. bulbocastanum* accession. Three of the accessions holding the *Sen1* flanking marker are known to be resistant to P1 (Supplementary File 3). With CoSSA, we observed that *Sen1* was found in only one accession (landrace PI 258885), belonging to the *S. tuberosum* species from the Chilotanum subgroup, which shared 63.3% of the *Sen1* specific *k*-mers mapping between 1.2 and 1.7 Mb on chromosome *11* (Supplementary File 6).

With the *Sen2* CAPS marker, we identified positive signals in four *Solanum acaule* accessions from the Acaulia series. Three accessions from the Demissum series were also positive for the *Sen2* marker (one *S. demissum* accession and two *S. guerreroense*). Finally, three accessions from the Tuberosa series (one *S. spegazzini* and two *S. verrucosum*) were positive for the *Sen2* marker (Fig. 4, Supplementary File 3, Supplementary Figure 13).

For *Sen3*, *Sen4* and *Sen5*, we observed that none of the screened accessions held both flanking markers, except for one *S. tuberosum* ssp. *andigena* accession which hold both *Sen3* flanking markers The *Sen3* flanking marker chr11_1259552 was present in 24 accessions, whereas the other flanking marker chr11_1772869 was present in only three accessions (Supplementary File 3). This second marker mapped closer to *Sen3* in the KxL population (Prodhomme et al. 2019) and was present in two *S. guerreroense* accessions (Demissa series) which have an intermediate level of resistance to P8, and the *S. tuberosum* ssp. *andigena* accession (Tuberosa 3 series). With CoSSA, *Sen3* was also found in a *S. tuberosum* landrace (PI 245847) from the Chilotanum subgroup. It shared 93.2% of the *Sen3* specific *k*-mers mapping between 1.2 and 1.8 Mb on chromosome *11* (Supplementary File 6).

The *Sen4* flanking markers chr12_48501410 and PotVar0037404 were found each in one accession (Supplementary File 3). In our AxV population, PotVar0037404 co-segregated with *Sen4*. It was found in a *S. circaelifolium* accession from the Circaelifolia series, known to have an intermediate level of resistance to P2 and P6. For *Sen4* with CoSSA, it was more difficult to determine if an accession held the gene or not as a smaller proportion of *k*-mers was shared in the wide genomic region identified. To determine if an accession held the *Sen4* gene, we compared the distribution pattern of *Sen4* specific *k*-mers mapped to each chromosome *12* bin between Axion and the tested accessions (Supplementary File 6). The *S. brevicaule* accessions PI 498112 and PI 545987 shared 42.6% and 43%, respectively, of the *Sen4* specific *k*-mers between 48.5 and 50.7 Mb and had a distribution pattern of mapped *k*-mers similar to Axion. It was also the case of four *S. tuberosum* group Andigena accessions (PI 245935, PI 245940, PI 546023 and PI 607886) and one *S. tuberosum* group Chilotanum (PI 245847) accession in which was found *Sen3* as well.

The *Sen5* flanking markers PotVar0035016, which was co-segregating with *Sen5* in AxD and KxA, and PotVar0034831, which was two recombination events from *Sen5*, were found in zero and 39 accessions, respectively (Supplementary File 3). PotVar0034831 was found in all the accessions from the Demissa and Acaulia series, nine accessions from the Tuberosa 3 series (including five *S. vernei* accessions), seven accessions from the North and Central American diploid series and two accessions from the Circaeifolia series (Fig. 4). Fourteen of these accessions are known to show an intermediate or strong level of resistance to at least one of the higher pathotypes (Supplementary File 3). *Sen5* was found with CoSSA in the *S. vernei* accession PI 473305 which contained 56.4% of the *Sen5* specific *k*-mers between 49.2 and 51.2 Mb (Supplementary File 6).

Regarding the minor effects QTLs, *Rse*-*Ib*-*Andante*-*a* was not found in any of the accessions, but chr01_76425362, present on both haplotypes a and c, was found in ten accessions, mainly from the Tuberosa series. *Rse*-*VIIIb*-*Kuba* was found in four accessions from the Tuberosa 3 series, including two accessions from *S. phureja* but also in the varieties Inca Sun and Mayan Gold, which are derived from *S. phureja* accessions. It remains to be shown if these varieties are resistant to *S. endobioticum* pathotypes*. Rse*-*IXb*-*Ludmilla* was identified in 67 accessions from various series and species. Interestingly, *Rse*-*XIc*-*VTN62.33.3* was found in three accessions from *S. vernei*. Finally, *Rse*-*XId*-*Andante* was identified in 17 accessions from various series and species.

## Discussion

### Identification of *Sen4*, important source of resistance in the Dutch breeding material

In this study, we performed a GWAS in the potato breeding genepool to identify markers linked with pathotypes 2, 6 and 18 resistance. The GWAS and the validation of the significant markers in a full-sib population allowed to identify the major effect locus *Sen4,* located on the south arm of chromosome *12,* linked with P2, P6 and P18 resistance. CoSSA was used to identify new markers flanking *Sen4* which could be mapped to a 3 Mb interval between 48.5 and 51.5 Mb. In this region of the reference genome, no large NLR or RLP or RLK clusters are found (Jupe et al. 2013; Nazarian-Firouzabadi et al. 2019). Only a single CNL gene (DMG401007575; Jupe et al. 2013) and a single RLK (StRLK66) were found as candidate genes. We found that *Sen4* was present in the variety Panda which was used as the resistant parent of the mapping population in the study of Groth et al. (2013). A QTL matching the *Sen4* locus was, however, not identified in their study which is probably due to the use of a limited number of SSR markers. In the BNA2 population from Obidiegwu et al. (2015), the locus *RSe*-*XIIa* from the central region of chromosome *12* was associated with P2, P6 and P18. The alternative allele of Solcap_c2_33630 was, however, present in many susceptible varieties and was present in duplex in the resistant parent (Karolin) and in the susceptible parent (unknown). For these reasons, it is unlikely that *RSe*-*XIIa* is the same locus as *Sen4*. Moreover, we tested Karolin which was negative for *Sen4*.

We observed that *Sen4* is Identical-By-Descent (IBD) in the Dutch varieties and could be traced back with markers to the breeding clone AM78-3704 and even further to its grand-parent Alcmaria. Alcmaria comes from a cross between Sirtema and a progeny from Saskia x (CPC 1673-20 × Furore) (van Berloo et al. 2007). We tested genome sequencing data from Sirtema for the chromosome *12* associated SNPs (data not shown) but could not find them. We concluded that Sirtema does not contain *Sen4.* Therefore, *Sen4* probably comes from the breeding clone CPC1673-20 which has Andigenum background. Interestingly, wart resistance had already been observed in CPC1673 which was used to introgress nematode resistance in the breeding genepool (Ross 1986). The only variety which holds *Sen4* not IBD from Alcmaria is Panda. Panda’s *Sen4* allele probably comes from a different source than the Dutch material. In the genotypic data from Uitdewilligen et al. (2013), we observed that the *Sen4* associated markers were present in the variety Hindenburg which is resistant to the pathotype 1 (Bukasov 1940) but whose resistance to the higher pathotypes is unknown. Hindenburg is present in the pedigree of Panda through the varieties Aquila and Mittelfruhe. We tested Aquila which was negative for the *Sen4* markers. Testing the Mittelfruhe pedigree branch with the *Sen4* markers may reveal if Hindenburg can be the German source of *Sen4*.

In the Solanum panel, the *Sen4* marker the most closely linked with resistance was present in one *S. circaeifolium* accession (Circaeifolia series). *S. circaeifolium* is known to contain resistance to other pathogens such as *P. infestans*, *G. pallida* and *E. carotovora* (Mattheij et al. 1992). This accession was phenotyped and found to be moderately resistant to P2 and P6 but susceptible to P8. In the panel from Hardigan et al. (2017), *Sen4* was found in two *S. brevicaule* accessions, a landrace from the group Chilotanum and several landraces from the *S. tuberosum* group Andigena. This last observation strengthens our hypothesis that the Dutch material source of *Sen4* might be the clone CPC1673 which possesses Andigenum origins.

### Identification of *Sen5*, underrepresented source of resistance in the breeding genepool

The two bulks from Prodhomme et al. (2020b) were re-used in an adapted CoSSA workflow to characterize the resistance from Aventra. We identified and mapped *Sen5* on the south arm of chromosome *5* between 49.2 and 51.16 Mb. In the reference genome, the C40 and C41 NLR clusters are located between 49.48 and 49.69 Mb (Jupe et al. 2013) and there are four gaps in this region in the reference genome (Fig. 3d). In the variety panel, *Sen5* was very rare as it was found in only two varieties (Aventra and Kanjer) and one breeding clone (VE71-105). A closer look to the pedigree showed that Kanjer is a descendant of VE71-105. The pedigree of Aventra is unknown, but we hypothesize that these three resistant genotypes are IBD for *Sen5*, sharing VE71-105 as the donor. The screening of the two flanking markers in the Solanum panel gave contrasting results as one marker was not found in any accession, and the other was found in 39 accessions including all the accessions from the Demissa and Acaulia series, accessions from the Tuberosa series (including several *S. vernei* accessions) and several accessions from North and Central America and the Circaeifolia series. As it is unlikely that *Sen5* is present in so many accessions from so many different phylogenetic series, we hypothesize that marker PotVar0034831 detects a more common SNP adjacent to the introgression segment of the *Sen5* donor haplotype. CoSSA gave less ambiguous results than the marker analysis, and *Sen5* was found in one *S. vernei* accession. This is in agreement with the observation that VE71-105 has multiple *S. vernei* sources in its pedigree (van Berloo et al. 2007). *S. vernei* has previously been reported as a potential source of wart disease resistance (Maris 1961).

### Diagnostic power of the *Sen1* flanking markers and origin of *Sen1*

Ballvora et al. (2011) identified in the SaKa1 population the *Sen1*-*XI* locus on the north arm of chromosome *11* which brings a quantitative resistance to P1. The authors suspected this locus to be an allelic variant of *Sen1* or a different *R* gene from the same cluster. However, the screening of the *Sen1* flanking markers from Prodhomme et al. (2020b) showed that *Sen1* is linked to P1 resistance in the SaKa1 population. In the study of Ballvora et al. (2011), the GP259 markers from the *Sen1* region were most associated with P1 resistance. The BLAST of GP259 to DM v4.03 showed this fragment is located between 2,805,301 bp and 2,805,774 bp which is more than 1 Mb from *Sen1*. This may explain why the *Sen1*-*XI* locus did not bring a clear qualitative resistance to P1, while in our studies the two flanking *Sen1* markers (chr11_1308927 and chr11_1666351) did detect qualitative P1 resistance.

*Sen1* was present in 60% of the varieties screened and as described previously (Prodhomme et al. 2020a), it is not possible to trace it back to a common ancestor donor. In the accessions panels, *Sen1* was found mainly in the diploid Tuberosa 3 series (Hawkes 1990) accessions originating from Bolivia and Argentina. More specifically, it was found in the *S. tuberosum* group Chilotanum, in *S. microdontum*, *S. vernei* and one accession from *S. bulbocastanum*. Members of the group Chilotanum, adapted to long days, are ancestors of commercial cultivars (*S. tuberosum* ssp. *tuberosum*) and contributed greatly to its genetic background. A high number of introgressions from *S. microdontum* have been found in long-day adapted cultivars (Hardigan et al. 2017). The intensive use of Chilotanum group and *S. microdontum* to adapt potato to long-days could have led to multiple introgressions of *Sen1* early in the group Tuberosum which would explain the lack of identity-by-descent (IBD) of *Sen1*. In Khiutti et al. (2012), the *Sen1* linked marker Nl25 (Hehl et al. 1999) was identified in two accessions from *S. tuberosum* ssp. *andigenum* and three accessions from *S. tuberosum* ssp. *tuberosum*. In this study, and in the broad panel of accessions screened for P1 resistance by van Soest and Seidewitz (1981), no correlation was found between the resistance to P1 and the taxonomy, the ploidy level or the geographic origin, which might be explained by the presence of *Sen1* very early in the ancestors of cultivated potato.

### Sporadic presence of *Sen2* in the breeding material

*Sen2* was identified for the first time in the complex diploid hybrid DG 97-264 (Plich et al. 2018). In our variety panel, none of the varieties showed a similar banding pattern compared to DG 97-264 except for Bonza which is known to be resistant to P1, P2 and P6. However, Bonza’s resistance might come from *Sen3* as it holds one of the two *Sen3* flanking markers. The pedigree of Bonza is unknown, so no hypothesis can be made about if Bonza hold a recombination in the *Sen3* haplotype. Most likely, *Sen2* has not yet been included in the breeding programs and is therefore a new source of wart disease resistance.

In the accessions panel, three accessions from the Demissum series (*S. demissum* and *S. guerreroense*), four *S*. *acaule* accessions, two *S. verrucosum* accessions and one *S. spegazzini* accession showed a banding pattern similar to DG 97-264. *S. acaule*, *S. demissum* and *S. verrucosum* have been used in the pedigree of DG 97-264 and either of them might be the resistance donor. *S. acaule* has been reported as being a potential source of wart disease resistance (Maris 1961) and more specifically as being the source of the P1, P6 and P18 resistance of Saphir through the donor MPI44.1016/24 (Ross 1986). We could, however, reject the hypothesis that Saphir or MPI 44.1016/24 were positive to the *Sen2* CAPS marker.

The scoring of the Sen2_CAPS marker was not unambiguous. In order to validate if variety Bonza and the accessions that we scored positive for *Sen2* indeed hold the same allele as the clone DG 97-264, we would recommend to sequence the *Sen2* marker PCR products. Another unambiguous way to verify the presence of *Sen2* in other sequenced varieties or accessions would be to identify *k*-mers specific to *Sen2*. This could be done by sequencing two bulks of resistant and susceptible descendants from the SEN 12-01 population (Plich et al. 2018) or by sequencing the PCR fragments which are linked with resistance in DG 97-264 and comparing their *k*-mers with other genotypes using CoSSA.

### *Sen3* is an important source of resistance in the German and Polish material

It has been shown previously that *Sen3* was IBD in all the *Sen3* positive varieties and could be traced back to the variety Ora, a descendant of Capella and BRA9089 (Prodhomme et al. 2019). In the old literature, resistance to the higher pathotypes was traced back to the clone BRA9089 which was found at that time to be resistant to P1 and to the higher pathotypes. In Prodhomme et al. (2019), BRA9089 was found to be susceptible to all pathotypes and negative for the *Sen3* flanking markers, which is most likely due to the fact that the old and recent BRA9089 clones are different. In our variety panel, *Sen3* was mainly found in German and Polish varieties. All these *Sen3* varieties for which the pedigree is complete hold BRA9089 in their pedigree. Surprisingly, the *Sen3* markers were also found in the American varieties Defender and Goldrush and in the Dutch variety Lady Sara (descendant of Defender). We did not find any information about the resistance of these varieties to the higher pathotypes, nor did we pursue phenotyping of these clones. These three varieties also hold BRA9089 in their pedigree, which makes it the putative unique donor of *Sen3* in the breeding genepool. BRA9089 originates from crosses involving a cultivar from Chiloe (*S. tuberosum* ssp. *tuberosum*), the cultivar Svitez and a landrace (van Berloo et al. 2007; Bukasov and Kameraz 1959; Ross 1986).

In our Solanum panel, only one of the Solanum accessions was positive for both flanking markers which can be explained by the fact that one or both of them are located outside the introgression segment. The closest marker to *Sen3* was found in the two *S. guerreroense* accessions and in an accession from the *S. tuberosum* group Andigena. This group has been reported before as being a source of potato wart disease resistance (Bukasov and Kameraz 1959; Ross 1986; van Soest and Seidewitz 1981). CoSSA gave more robust results and clearly showed the presence of both *Sen3* and *Sen1* in one landrace from *S. tuberosum* group Chilotanum, showing that *Sen1* and *Sen3* were already present in this ancestral landrace of commercial cultivars.

### *Rse*-*Ib*-*Andante* shows co-dominant resistance to pathotypes 1, 2, 6 and 18

Ballvora et al. (2011) identified several alleles on the south arm of chromosome *1* linked with P1, P2, P6 and P18 resistance or susceptibility in SaKa1. *Sen2/6/18_a* linked with resistance is the same as *Rse*-*Ib*-*Andante*-*a* that we identified. In the accession panel, this haplotype was not identified in any of the accessions tested (Supplementary File 3). The haplotype *Sen2/6/18*-*_b* linked with susceptibility is the same as *Rse*-*Ib*-*Andante*-*b*. We identified an extra haplotype at the *Rse*-*Ib*-*Andante* locus: *Rse*-*Ib*-*Andante*-*c* is one of the two alleles on which the marker chr01_76425362 is present. This marker was identified in ten accessions from the panel, seven of which belong to the Tuberosa series. With CoSSA, we mapped *Rse*-*Ib*-*Andante* to the south arm of chromosome *1* between 70 and 79.6 Mb. In the reference genome, this region contains the two NLR clusters C4 and C5 (Jupe et al. 2013) and the four RLK genes *StRLK04*, *StRLK05*, *StRLK06* and *StRLK07* (Nazarian-Firouzabadi et al. 2019). Interestingly, the two CNL genes from the cluster C4 are homologues of NRC1, a helper NLR which is required by cell surface and intracellular immune receptors (Gabriëls et al. 2007). The involvement of different alleles of this helper gene in resistance to wart disease mediated by *Rse*-*Ib*-*Andante* might explain the quantitative, genetic background-dependent effect of *Rse*-*Ib*-*Andante*. The different resistance levels induced by *Sen* genes, which could serve as sensor NLRs, would then depend on the NLR helper they can pair with.

### Identification of minor effect potato wart disease resistance QTLs

In the three populations AxV, AxD and KxA, we identified *Rse*-*XIc*-*VTN62.33.3*, which brought a quantitative resistance to pathotypes 2, 6 and 18. The recognition pattern of this haplotype depended on the population in which it segregated and on the phenotyping assay that was used: *Rse*-*XIc*-*VTN62.33.3* was associated with P2 and P6 resistance in AxV, with P6 and P18 in AxD and with P2 and P6 (Glynne–Lemmerzahl) and P18 (Spieckermann) in KxA. One reason for these differences could be that different isolates were used in the different tests. This could be the reason for the difference between the AxV and the AxD/KxA populations, but not for the difference between AxD and KxA which were phenotyped with the same isolates. A second explanation could be that several genes underlie this QTL and that recombinations happened between them in some of the populations. A third explanation could be a background dependency of this QTL. *Rse*-*XIc*-*VTN62.33.3* was mapped to the 5 first Mb of the north arm of chromosome *11*. This broad region of chromosome *11* contains the three NLR genes clusters C76, C77 and C78 (Jupe et al. 2013), two RLK genes and one RLP (Nazarian-Firouzabadi et al. 2019). This haplotype was identified in four *S. vernei* accessions from the panel. *Rse*-*XIc*-*VTN62.33.3* was probably introgressed in the breeding genepool through the *S. vernei* ancestor of the VTN62-33-3 breeding clone.

In the SaKa1 population, Ballvora et al. (2011) identified the *Sen18*-*IX* locus bringing resistance to P18, located on the south arm of chromosome *9.* We also identified this locus in our study, and renamed it *Rse*-*IXa*-*Andante* to comply with the new naming system we introduced in this study. We mapped *Rse*-*IXa*-*Andante* between 55 and 61.5 Mb on chromosome *9*. This region is rich in NLR genes as it contains the C64, C65 and C66 clusters (Jupe et al. 2013), and four RLK genes (Nazarian-Firouzabadi et al. 2019). Another minor effect QTL for pathotypes 2, 6 and 18 from the variety Ludmilla was identified at this locus by Prodhomme et al. (2019). The Ludmilla haplotype was different from *Rse*-*IXa*-*Andante*, as determined using CoSSA, so we renamed it *Rse*-*IXb*-*Ludmilla* to avoid confusion and to comply with our updated naming system. The marker from this haplotype screened in the accession panel was found in 67 accessions. This high frequency can probably be explained by the presence of the marker outside the introgression segment.

In the SaKa1 population, we identified an extra haplotype linked with P2, P6 and P18 on the north arm of chromosome *11* which was not identified previously. *Rse*-*XId*-*Andante* is different from *Rse*-*XIc*-*VTN62.33.3* identified in the AxV, AxD and KxA populations, as determined using CoSSA. With CoSSA, we mapped *Rse*-*XId*-*Andante* to a very broad region between 0 and 31 Mb. The marker from this haplotype was identified in 17 accessions from the panel, no link could be made with a specific species or series.

In the KxL population (Prodhomme et al. 2019), we identified *Rse*-*VIIIb*-*Kuba*, a minor effect locus on the south arm of chromosome *8* which improved the resistance to P18 brought by *Sen3*. The two SNPs linked with P18 resistance identified with CoSSA (chr08_44797542 and chr08_45178832) are located in a region rich in NLR gene clusters as it contains the three NLR clusters C59 (45.03–45.12 Mb), C60 (47.54–48.15 Mb) and C61 (48.37–48.6 Mb) (Jupe et al. 2013). This haplotype was identified in four accessions from the Tuberosa 3 series.

### Do major and minor effect loci complete the resistance spectrum to cover intra-isolate diversity?

In this study, we observed that the stacking of major and minor effect loci was increasing the resistance levels to potato wart disease. In the AxV population, the presence of *Sen4* and *Rse*-*XIc*-*VTN62.33.3* improved the resistance to P2 and P6. Similarly in the AxD and KxA populations, the stacking of *Sen5* and *Rse*-*XIc*-*VTN62.33.3* improved resistance to P2, P6 and P18. In the SaKa1 population, the stacking of *Rse*-*Ib*-*Andante* and *Rse*-*XId*-*Andante* for P2 and P6 and of *Rse*-*IXa*-*Andante* for P18 was required to give strong resistance. Finally, in the KxL population, we observed that the presence of *Rse*-*VIIIb*-*Kuba* improved the resistance to P18 brought by *Sen3.* An explanation for the additive effect of minor- to major effect loci in potato wart disease resistance can be sought in the genotypic diversity within *S. endobioticum* isolates used for the phenotypic assays (van de Vossenberg et al. 2018). For instance, in the case of the KxL population, we can hypothesize that *Sen3* is a TNL that recognizes an effector which is present in all genotypes of the P2 and P6 isolates and in the majority of the genotypes of the P18 population. In concert, *Rse*-*VIIIb*-*Kuba* might be a CNL that recognizes an effector which is in a minority of the P18 isolate population. A similar explanation could hold for *Rse*-*Ib*-*Andante*-*a* and -*c*, *Rse*-*IXa*-*Andante*, *Rse*-*XId*-*Andante* and *Rse*-*XIc*-*VTN62.33.3* which also co-localize with NLR clusters. Overall, the stacking of several loci to bring full resistance seems to be essential for higher pathotypes. The higher intra-isolate diversity observed in isolates from higher pathotypes such as P8, P18 and P38 would indeed explain why resistance to the higher pathotypes is more quantitative (van de Vossenberg et al. 2018).

### Unknown resistances remain in the breeding genepool

There are resistances present in the potato breeding germplasm which do not come from any of the identified *Sen* genes and are, therefore, still unknown. It is the case for instance, of the P2, P6 and P18 strong resistance of Belita which probably comes from *S. vernei* through the clone VTN62-33-3, donor of *G. pallida* resistance, or through the clone VTN61-13-9 (van Berloo et al. 2007). However, when we phenotyped VTN62-33-3, it turned out to be susceptible to P2, P6 and P18. The resistance to P6 and P18 present in Saphir is also not caused by *Sen1 2*, *3*, *4*, or *5*. This resistance likely comes from the breeding clone MPI 44.1016/24, which shows strong resistance to pathotypes 1, 2, 6 and 18, and *S. acaule*, as it was hypothesized by Ross (van Berloo et al. 2007; Ross 1986).

### The EPPO set does not encompass the *R* loci diversity present in the potato breeding genepool

The EPPO set is composed of differential potato varieties which are used for the identification of *S. endobioticum* pathotypes (EPPO 2017). The varieties from the EPPO set resistant to P1 contain either *Sen1*, or *Sen3*. *Sen1* is a TNL gene (Prodhomme et al. 2020b) which triggers a hypersensitive response (HR) upon the recognition of the *S. endobioticum* effector AvrSen1 present in the pathotype 1 isolates (van de Vossenberg et al. 2019). *Sen3* is also located in a TNL cluster, and we suspect it to bring resistance to P1 and P8 in addition to P2, P6 and P18 (Prodhomme et al. 2019) as it is present in several varieties resistant to P1 that do not contain *Sen1* and in several varieties resistant to P8. Moreover, we suspect that the chromosome *11* locus segregating in the BNA2 population is *Sen3*, and it was causing P1 resistance in addition to P2, P6 and P18 in BNA2 (Obidiegwu et al. 2015). *Sen2*, *Sen3*, *Sen4* and *Sen5* are not represented in the EPPO set. *Sen5* specifically recognizes P2, P6 and P18 isolates and not P1 isolates. *Sen4* seems to provide resistance to at least P6 and P18 as illustrated by the resistance spectrum of the variety Celandine. This spectrum was also supported by the GWAS study as *Sen4* SNPs were associated with P6 and 18 resistance but not with P1 and P2 resistance. The resistance spectrum of Panda suggests that the *Sen4* resistance spectrum is broader. Also in our populations studies (AxV), we saw association of *Sen4* with P2 resistance. Maybe *Sen4*, in association with minor QTLs, covers the intra-isolate diversity of other pathotypes as well. Another, more trivial explanation could be the ambiguity of the outcome of the Glynne–Lemmerzahl and Spieckermann tests. The first assay uses fresh warts and the second uses composted wart material and consequently, the summer and winter spores, respectively, contribute stronger to the infection process. It remains to be established if there is a difference in expression of effector proteins in infectious material derived from two types of spores. *Sen2* and *Sen3* can recognize a broad spectrum of pathotypes (P1, P2, P6, P8 and P18). For *Sen2,* more pathotypes were tested and showed to provide also P3 and P39 resistance (Plich et al. 2018). The inclusion of varieties holding the *Sen2*, *Sen4* and *Sen5* genes in the EPPO set will lead to the identification of additional *S. endobioticum* diversity and might lead to a higher resolution of pathotype identities. Moreover, it will give direct information to inform about the resistances and varieties for quarantine deployment. As discussed previously, there are also varieties from the EPPO set for which the resistance loci have not been identified. This is the case for Talent, Delcora, Saphir, Miriam and Belita. For a more complete understanding of pathogen diversity, it is important to better characterize and update the EPPO set and base it on the *R* loci content of the varieties and the matching effectors they recognize.

### CoSSA is more robust than molecular markers for the screening of specific loci

In this study, we used two different approaches to screen panels of varieties and Solanum accessions for the five *Sen* resistance genes. The first approach was a screening using PCR markers flanking the *Sen* genes. This approach was successful and efficient to screen the variety panel but not the wild accessions. This was not due to the different ploidy levels of these accessions as the clustering in the KASP markers output allows to mix different ploidy levels. The most probable reason is that one or all the flanking markers designed were not close enough to the resistance gene. If the SNP is not located on the introgression segment but on the *S. tuberosum* haplotype which recombined with the introgression segment, the SNP will not be linked with resistance in a panel composed of wild species and will lead to false positives. In the second approach, we used sequencing data and *k*-mers specific to the resistant haplotypes to verify the presence of the *Sen* genes. The results were unambiguous for *Sen1*, *Sen3* and *Sen5*. We could visualize the chromosome bins of the tested varieties/accessions which contained the resistance-specific *k*-mers. This visualization allows to determine unambiguously if the (fine-)mapped resistance locus is present in the tested genotype and it even allows to detect recombination events. This is the case for instance for *Sen1*. In Supplementary File 6, we can observe some varieties such as Atlantic (susceptible to P1), Kalkaska, Missaukee and Kennebec (susceptible to P1) which hold only the south part of the *Sen1* haplotype between 2.4 and 4.6 Mb. It is this part of the *Sen1* haplotype which was identified in the pathotype 1 GWAS study of Prodhomme et al. (2020a) as no markers located on the northern part of this haplotype, closer to the *Sen1* gene, were present on the 20 K Infinium array. The recombination event in Atlantic and Kennebec is visible in the Supplementary File 1 of the GWAS study between PotVar0067008 and solcap_snp_c1_2314, PotVar0106272, PotVar0106247 and PotVar0105904, and showed that *Sen1* was no longer present. For *Sen4*, it was more difficult to determine the presence of the gene in the CoSSA output because the *Sen4* specific *k*-mers peak was very broad (between 10.3 and 54 Mb) because insufficient recombinants were present in the resistant bulk.

## Conclusion

In this study, two new dominant major effect wart resistance genes, *Sen4* and *Sen5,* bringing resistance to higher pathotypes of *S. endobioticum*, were identified and mapped. Several minor effect QTLs were also identified that are necessary to bring a full resistance to certain pathotypes. Panels of varieties and Solanum accessions were screened for the presence of the *Sen* genes and QTLs. *Sen1*, highly frequent in the commercial varieties, was observed in a landrace from the *S. tuberosum* group Chilotanum and in several accessions from *S. microdontum* which might explain the presence of *Sen1* very early in the commercial varieties and the lack of identity-by-descent due to multiple introgressions. We suspect *Sen2* to be present in accessions from the Demissum series, *S. acaule*, *S. verrucosum* and/or *S. spegazzini. Sen3* could be traced back to the clone BRA9089 and is IBD in all the varieties from commercial germplasm. It was also observed in the Chilotanum group. *Sen4*, identical-by-descent in the Dutch varieties, could be traced back to the variety Alcmaria and was probably introduced in commercial varieties through the Andigena group and the breeding clone CPC1673-20 used to breed for nematode resistant material. *Sen5* is very rare in the commercial varieties. It could be traced back to the breeding clone VE71-105 and the wild species *S. vernei.*

## Electronic supplementary material

Below is the link to the electronic supplementary material.

Supplementary material 1 (DOCX 9841 kb)

**Supplementary File 1** Compilation of phenotypic data for GWAS analysis. Overview of the historical sources that provided phenotypic data on wart disease resistance in the potato varieties used in our GWAS panels. These sources include documents for National listing where VCU data were recorded, marketing brochures from commercial breeders as well as scientific publications. Several sources use different scales to describe the level of wart disease resistance. Quantitative scales were harmonised to a common scale ranging from 1 = susceptible to 10 = resistant. Qualitative data R and S were converted into 10 and 1, respectively. This allowed compilation of the second tab sheet showing all records used to calculate a single phenotypic score using REML. The third, fourth and fifth tab sheets contain the genotypic data of the P2, P6 and P18 GWAS panels. (XLSX 13284 kb)

**Supplementary File 2** Phenotypic and genotypic data of the five full-sib populations used in this study. Each Excel sheet comprises the phenotypic data of all the tests performed, the genotypic data of all the markers screened and information about the bulks composition. (XLSX 137 kb)

**Supplementary File 3** Variety and Solanum panels. For each variety/accession, the species, the ploidy, the year and the origin are given. For the accessions, the series (according to Hawkes 1990) are also given. The genotypic data of the markers screened in the two panels and the CoSSA results in the panel of varieties from Hardigan et al. (2017) are given. When resistance to P1, P2, P6, P8 or P18 is known, it is indicated (R = resistant, I = intermediate, S = susceptible). For the varieties positive for the *Sen3*, *Sen4* or *Sen5* markers, it is indicated if BRA9089, Alcmaria or VE71-105, respectively, are present in their pedigree (van Berloo et al. 2007). (XLSX 52 kb)

**Supplementary File 4** Whole genome sequencing data and analysis. Sequencing depths, a description of the different CoSSA workflows and a summary of the CoSSA *k*-mers subsets for all the workflows. (XLSX 23 kb)

**Supplementary File 5** Marker validation in biparental populations. Validation of the GWAS SNPs in the AxV population (sheet 1), validation of the GWAS and CoSSA SNPs in the AxD population (sheet 2) and in the KxA population (sheet 3), validation of the CoSSA SNPs in the SaKa1 population (sheet 4) and in the KxL population (sheet 5). (XLSX 25 kb)

**Supplementary File 6** CoSSA in Solanum accessions. Occurrence of *Sen* genes specific *k*-mers in the panel from Hardigan et al. (2017) using CoSSA. ^**a**^ Proportion of the *Sen* genes specific *k*-mers shared between the positive control and the variety/accession from Hardigan et al. (2017). The positive controls for *Sen1*, *Sen3*, *Sen4* and *Sen5* are, respectively, Desiree and Kuras, Kuba, Axion and Aventra. In bold are indicated the accessions that we consider containing the *Sen* gene according to this table and the extra sheets. Extra-sheets: *K*-mers specific to each of the four *Sen* genes (*Sen1*, *Sen3*, *Sen4* and *Sen5*) present in the varieties and accessions of Hardigan et al. (2017) mapped to the potato reference genome. The chromosomes were cut in bins of 100 Kb, and the number of *Sen* specific *k*-mers per bin was counted. The varieties positive for a specific *Sen* gene are highlighted in green. The (fine)-mapped region of each *Sen* gene is highlighted in yellow. (XLSX 704 kb)
