# Supplementary material for: A Hitchhiker’s guide to the potato wart disease resistance galaxy
Source: Theor Appl Genet. 2020 Sep 12;133(12):3419–39. doi: 10.1007/s00122-020-03678-x (PMC7567731; doi:10.1007/s00122-020-03678-x)
Supplement: Supplementary file 1 — Supplementary material 1 (DOCX 9841 kb) [file 122_2020_3678_MOESM1_ESM.docx]

# Supplementary Figures


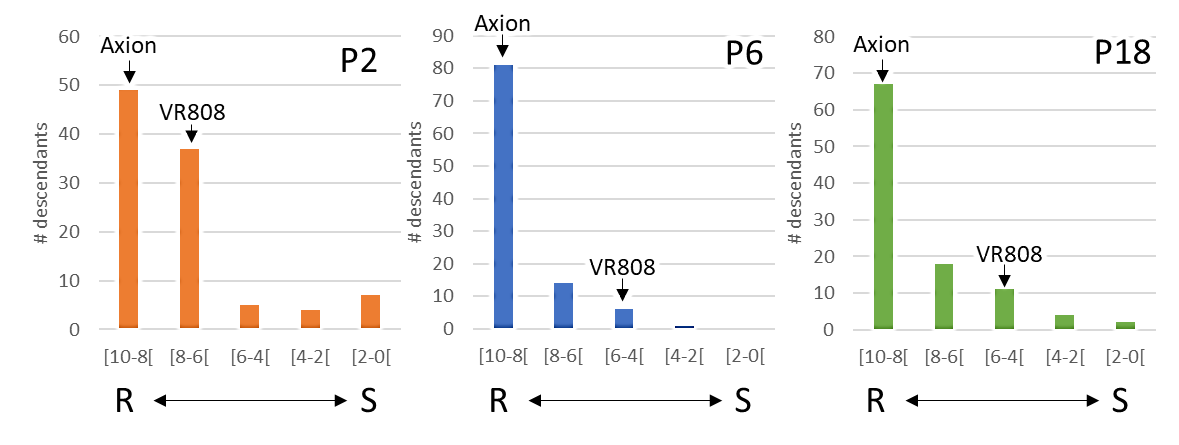


**Supplementary Figure 1**

Distribution of P2, P6 and P18 resistance assessed with the Spieckermann method in the AxV population. The resistance to the three pathotypes is skewed toward resistance. Quantitative scale: 10 = strongly resistant, 1 = strongly susceptible. The parents of the population are indicated.


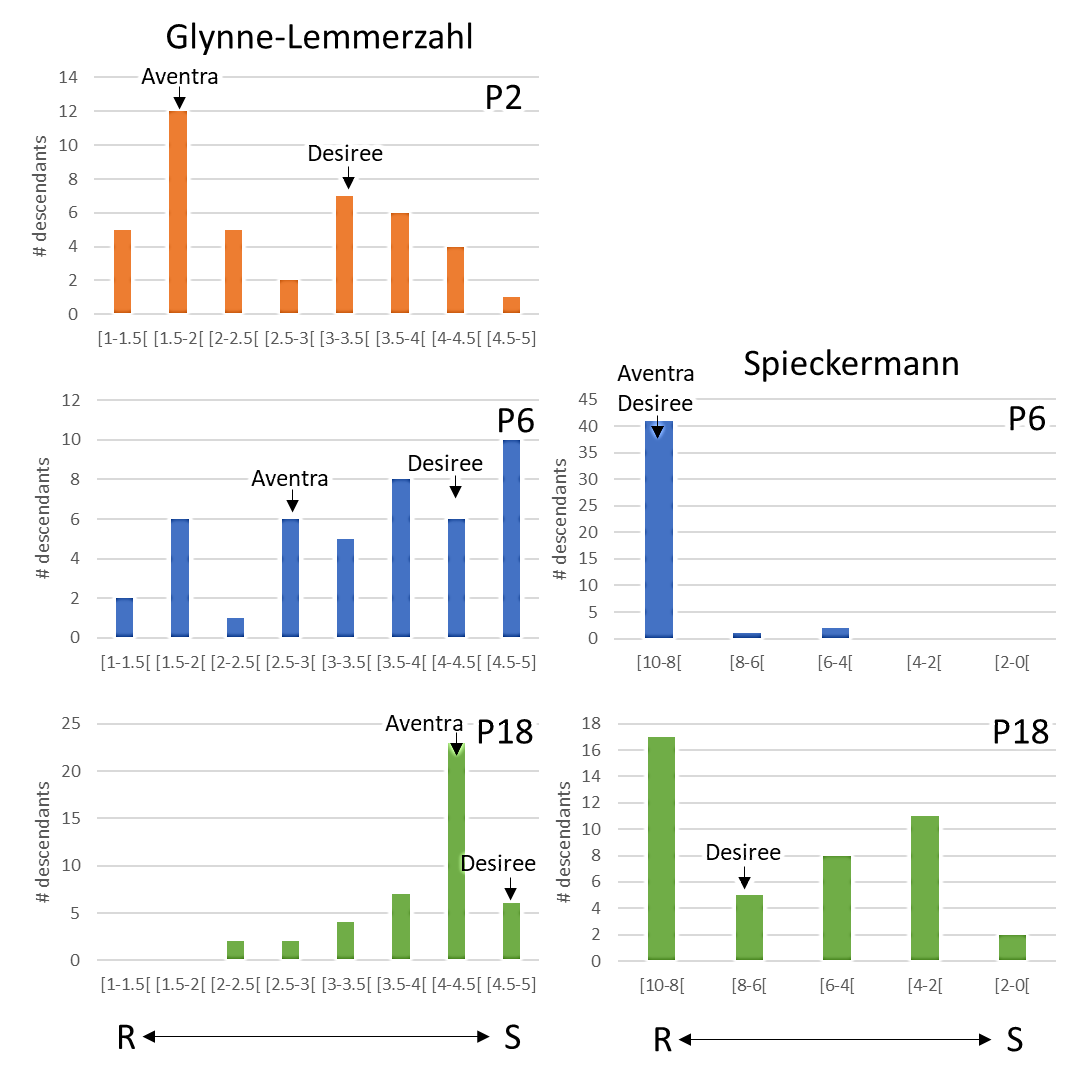


**Supplementary Figure 2**

Distribution of P2, P6 and P18 resistance assessed with the Glynne-Lemmerzahl method and of P6 and P18 with the Spieckermann method in the AxD population. Resistance to P2 showed a clear 1:1 bimodal distribution (Χ^2^ = 0.1, *p*-value = 0.75) whereas resistance to P6 did not show a clear 1:1 segregation pattern and resistance to P18 was quantitative, skewed toward susceptibility. This was not surprising since the resistant parent Aventra did not show a strong resistance (score of 4.0) to P18 with the Glynne-Lemmerzahl method either. With the Spieckermann method, 93% of the AxD descendants showed a resistance score of 8 or higher, suggesting a high number of escapes due to low virulence of the P6 isolate and the resistant scores were disregarded for further analyses. Fortunately, bimodal distribution of the resistance was observed with the Spieckermann method for P18 (Χ^2^ = 1.52, *p*-value = 0.217). Quantitative scale for the Glynne-Lemmerzahl scores: 1 = strongly resistant, 5 = strongly susceptible. Quantitative scale for the Spieckermann scores: 10 = strongly resistant, 1 = strongly susceptible. The parents of the population are indicated when phenotyped.


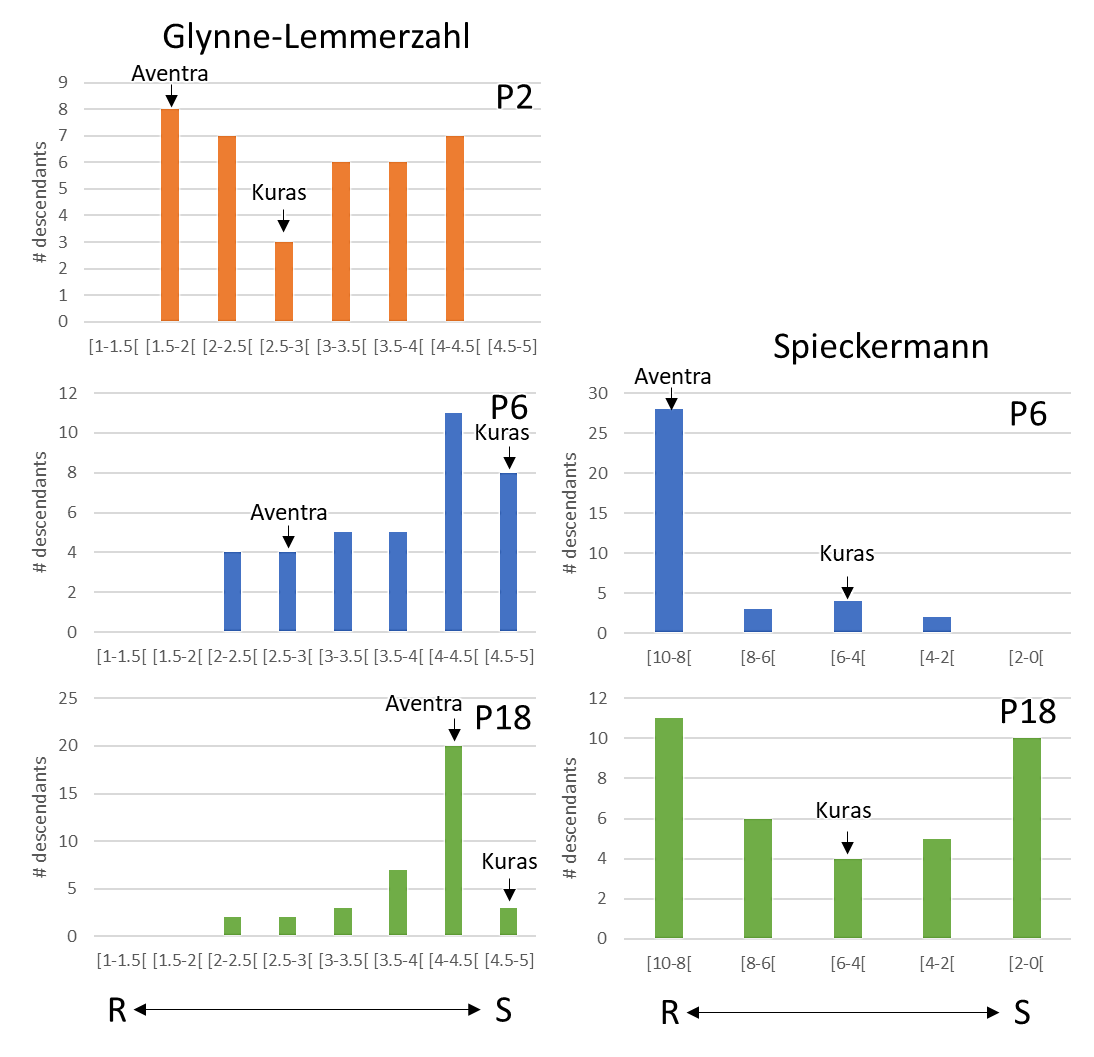


**Supplementary Figure 3**

Distribution of P2, P6 and P18 resistance assessed with the Glynne-Lemmerzahl method and of P6 and P18 with the Spieckermann method in the KxA population. With the Glynne-Lemmerzahl method, resistance to P2 was bimodal without any extreme phenotype (Χ^2^ = 1.4, *p*-value = 0.2367). Resistance to P6 and P18 was quantitative, skewed toward susceptibility. With the Spieckermann method, resistance to P6 was skewed toward resistance with 74.3% of the descendants scored with an 8 or higher. Resistance to P18 was bimodal (Χ^2^ = 0.029, *p*-value = 0.87). Quantitative scale for the Glynne-Lemmerzahl scores: 1 = strongly resistant, 5 = strongly susceptible. Quantitative scale for the Spieckermann scores: 10 = strongly resistant, 1 = strongly susceptible. The parents of the population are indicated when phenotyped.

**
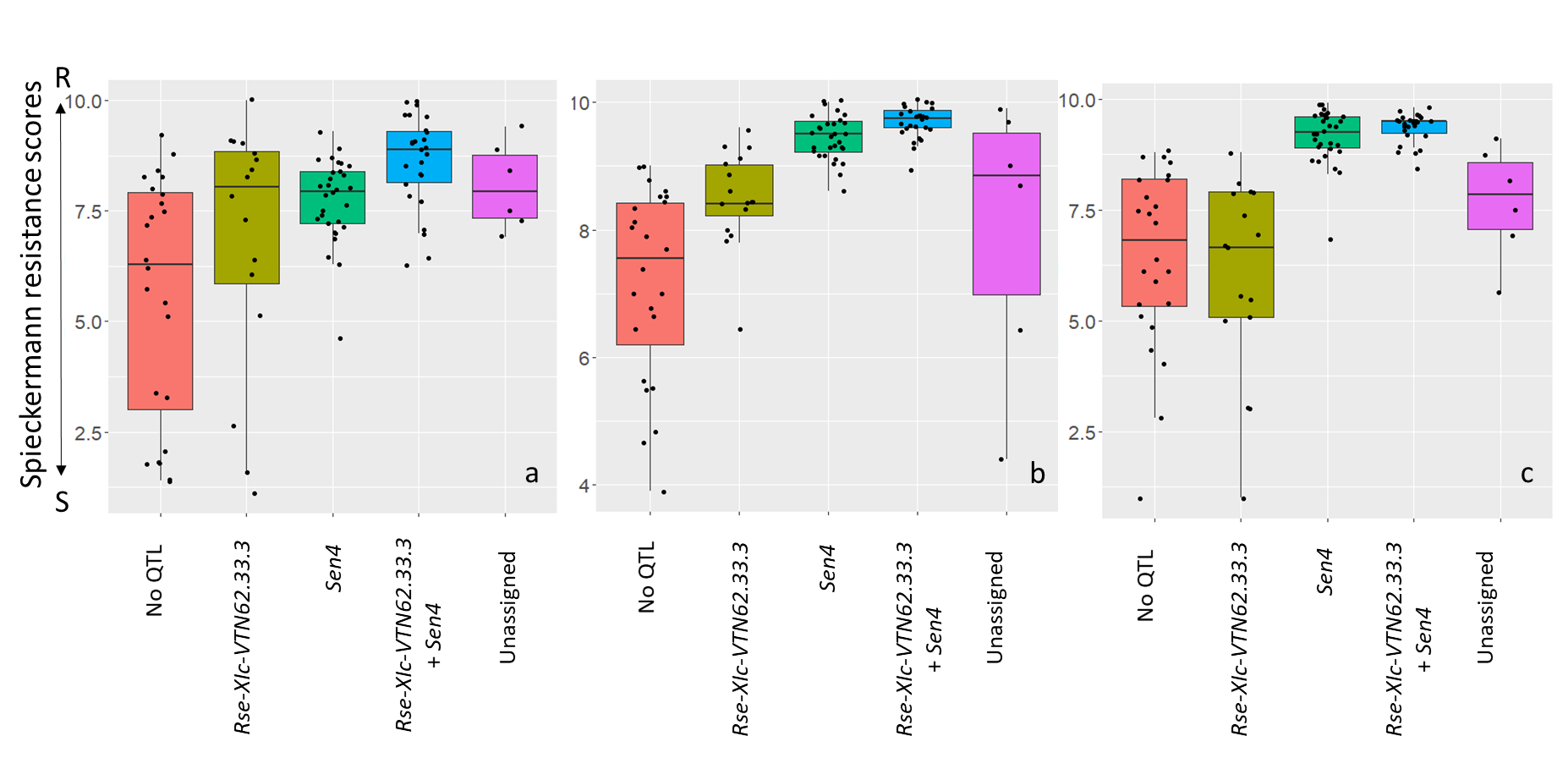
Supplementary Figure 4**

Effect of *Rse-XIc-VTN62.33.3* (PotVar0066337) and of *Sen4* (PotVar0037666) on **a** P2, **b** P6 and **c** P18 resistance in the AxV population.


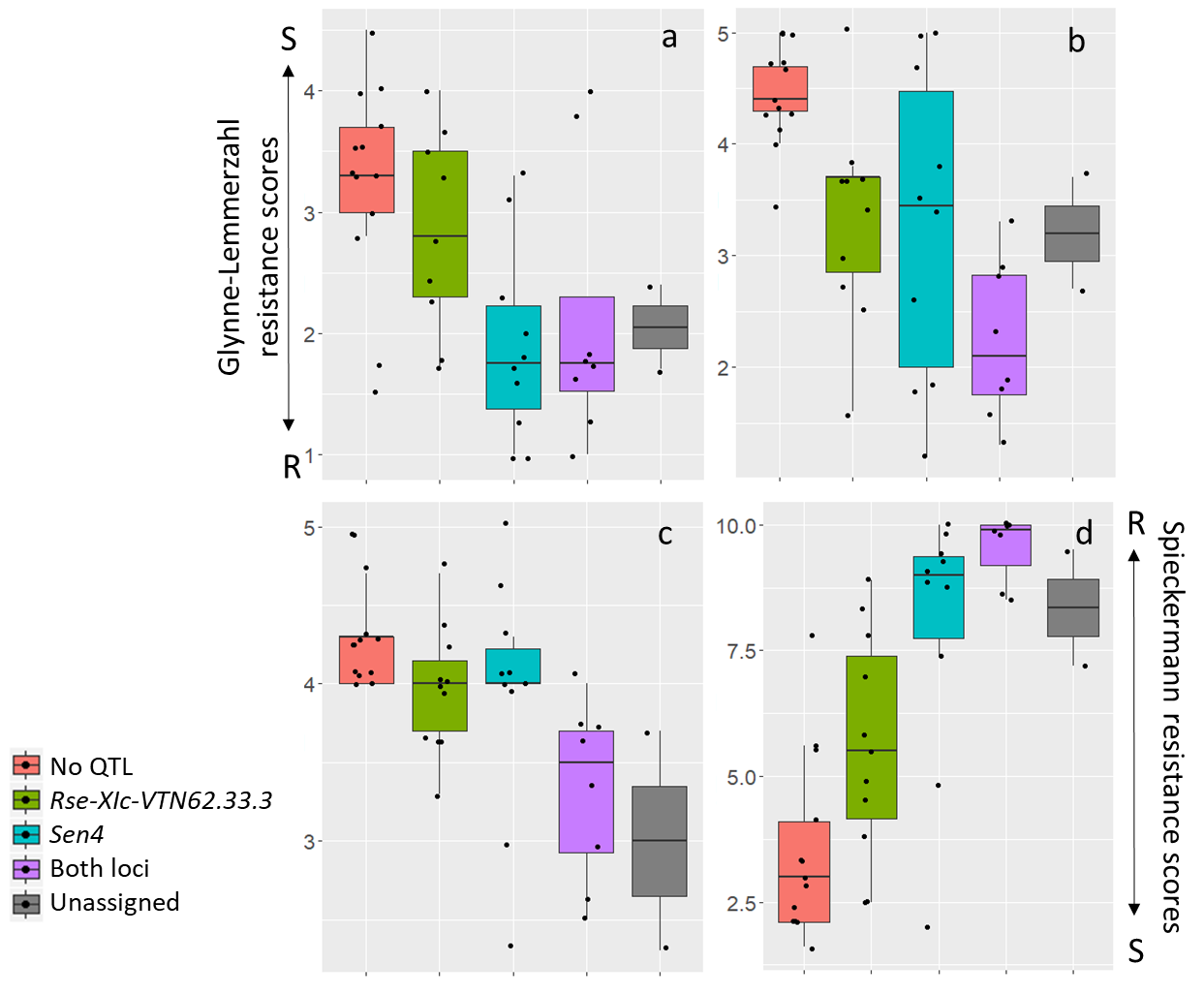


**Supplementary Figure 5**

**
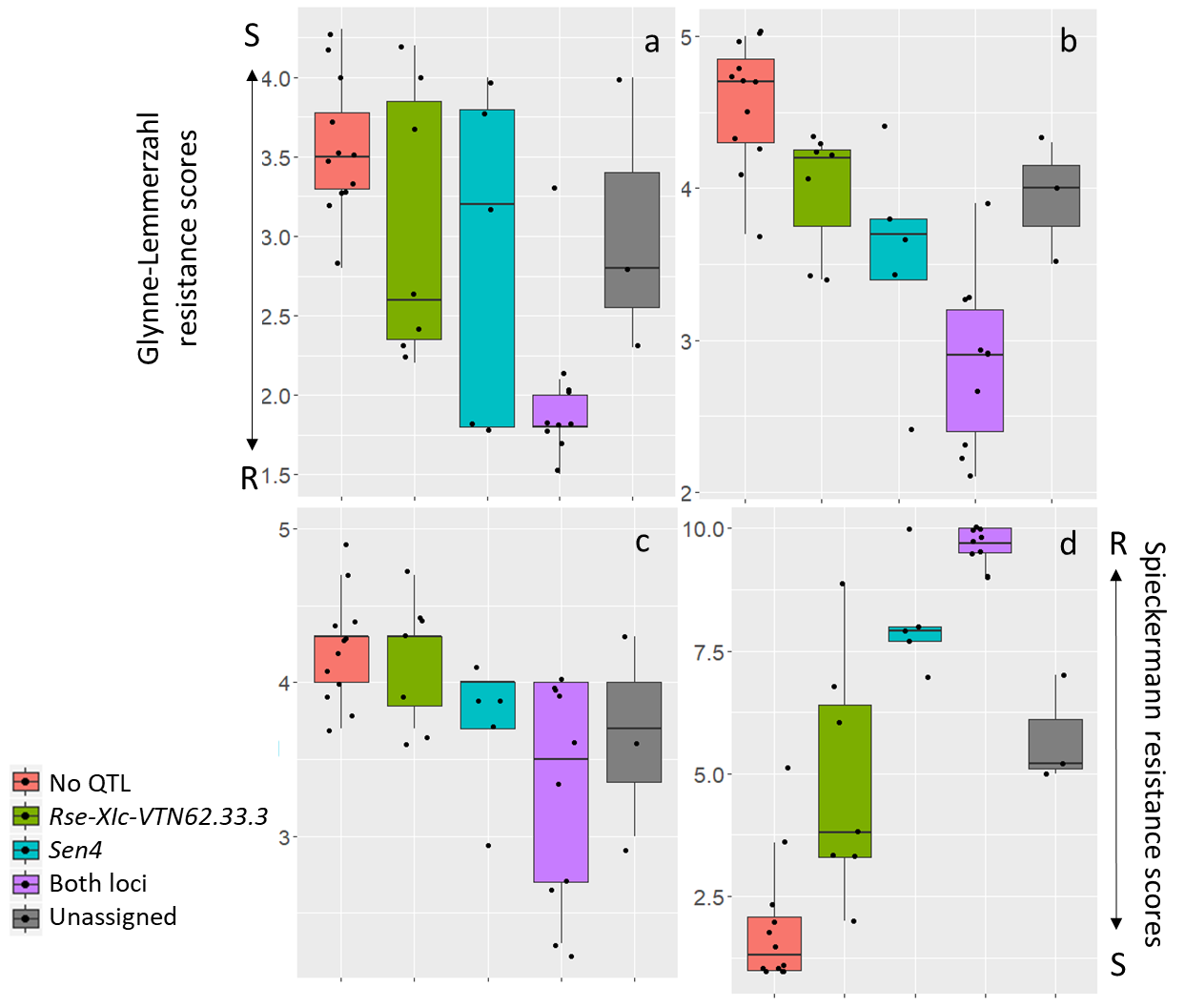
**Effect of *Rse-XIc-VTN62.33.3* (PotVar0067459) and of *Sen5* (presence of both flanking markers chr05_47980695 and chr05_52004982) on **a** P2, **b** P6, **c** P18 resistance assessed with the Glynne-Lemmerzahl method and on d P18 resistance assessed with the Spieckermann method in the AxD population.

**Supplementary Figure 6**

Effect of *Rse-XIc-VTN62.33.3* (PotVar0067459) and of *Sen5* (presence of both flanking markers chr05_47980695 and chr05_52004982) on **a** P2, **b** P6, **c** P18 resistance assessed with the Glynne-Lemmerzahl method and on d P18 resistance assessed with the Spieckermann method in the KxA population.

**
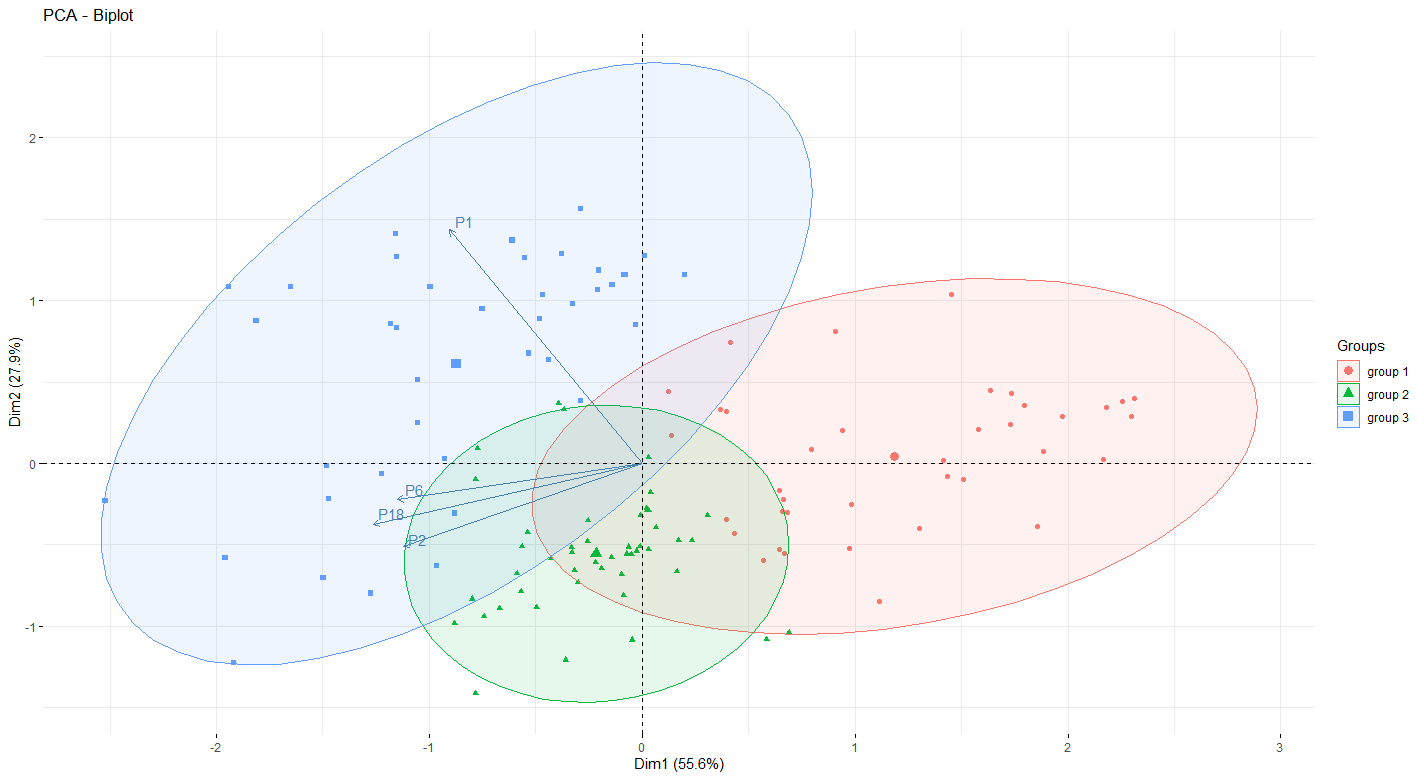
**

**Supplementary Figure 7**

Principal Component Analysis performed in SaKa1 on the resistance to P1, P2, P6 and P18. The two first principal components explained 83,5% of the variance. Three groups of descendants are visible: group 1 contains descendants resistant to P1, P2, P6 and P18, group 2 contains descendant resistant to P1 and group 3 contains descendant susceptible to all pathotypes.


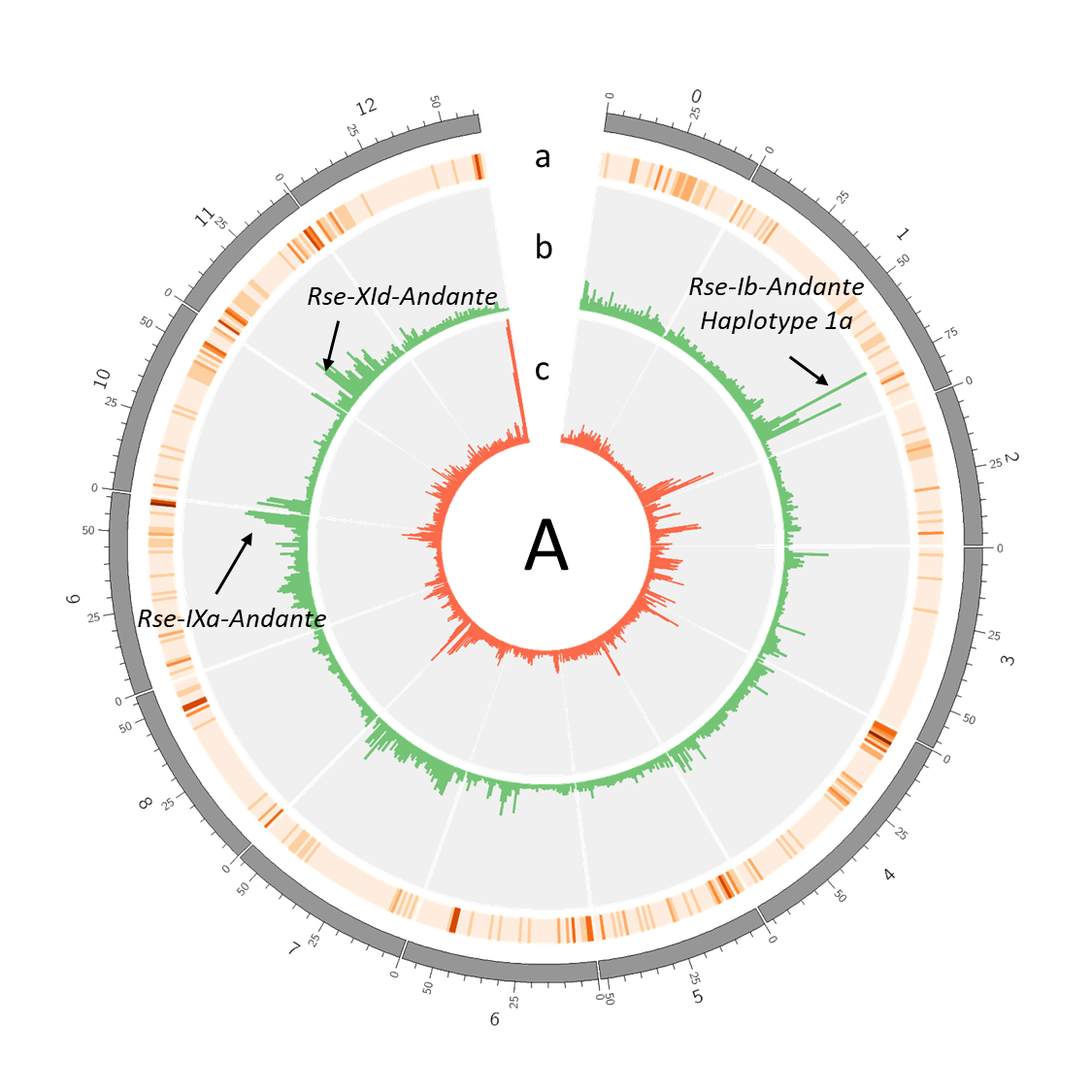


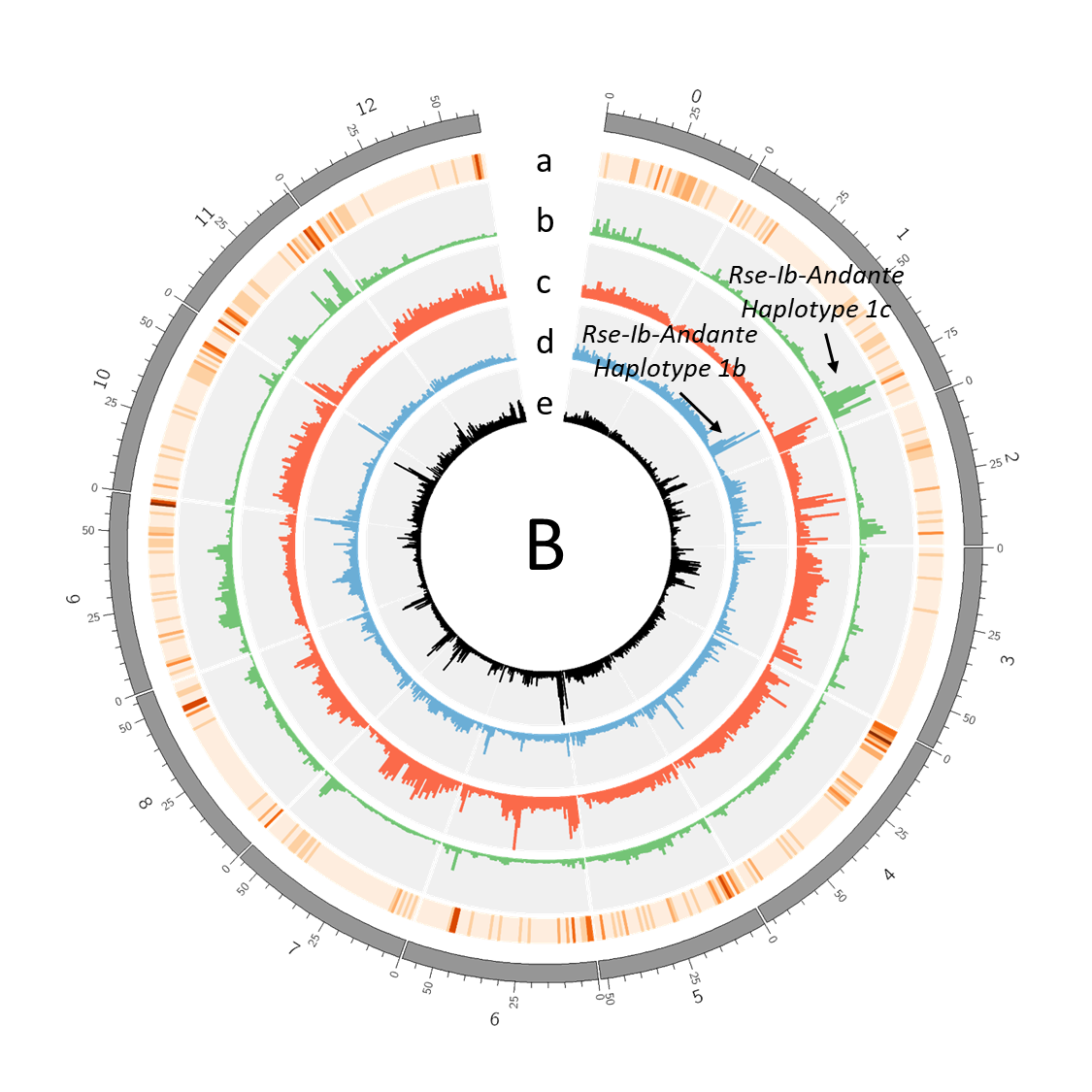


**Supplementary Figure 8**


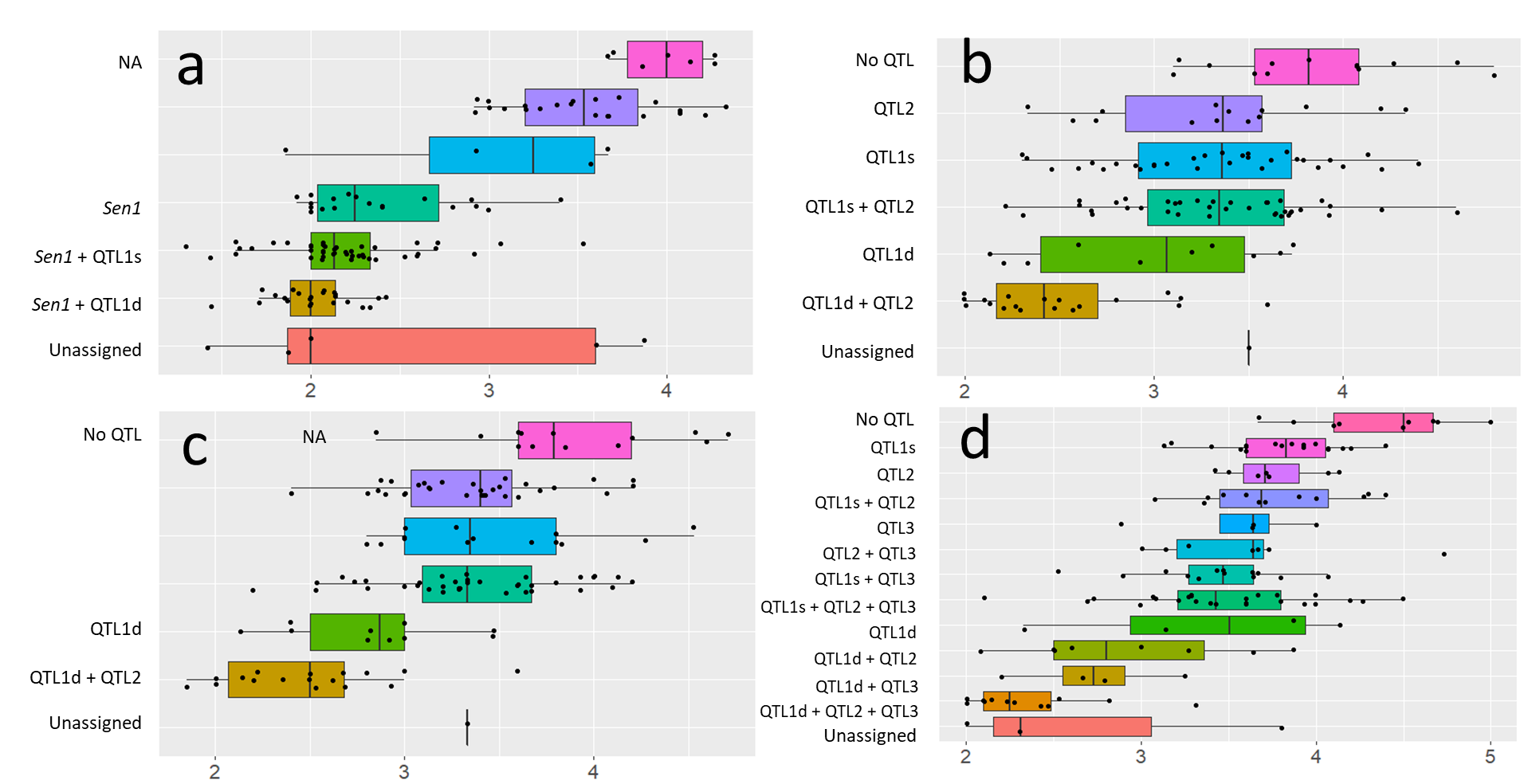
Comparative Subread Sets analysis in the SaKa1 population. **Aa** and **Ba** Number of NLR genes per bin of 1 Mb from the potato reference genome DM v4.03 according to (Jupe *et al*., 2013). **Ab** The SaKa1_RB bulk specific *k*-mers inherited from Andante mapped to the reference genome (y_max_ = 661%). **Ac** The SaKa1_RB bulk specific *k*-mers inherited from Alegria mapped to the reference genome (y_max_ = 545%). **Bb** The SaKa1_chr01_SB specific *k*-mers inherited from Andante mapped to the reference genome (y_max_ = 356%). **Bc** The SaKa1_chr01_SB specific *k*-mers inherited from Alegria mapped to the reference genome (y_max_ = 250%). **Bd** The SaKa1_chr01_RB specific *k*-mers inherited from Andante mapped to the reference genome (y_max_ = 321%). **Be** The SaKa1_chr01_RB specific *k*-mers inherited from Alegria mapped to the reference genome (y_max_ = 292%). For each chromosome, the average of the number of R-bulk specific *k*-mers mapped has been calculated and for each 1 Mb bin, the number of *k*-mers mapped from each set has been transformed to a percentage of the chromosome average.

**Supplementary Figure 9**

Effect of the QTLs identified in SaKa1 on **a** P1, **b** P2, **c** P6 and **d** P18 resistance. QTL1s = *Rse-Ib-Andante* haplotype 1a in one copy (chr01_76425362 in simplex), QTL1d = *Rse-Ib-Andante* 1a in two copies (chr01_76425362 in duplex), QTL2 = *Rse-XId-Andante* (chr11_11859618), QTL3 = *Rse-IXa-Andante* haplotype from Andante (chr09_61085336).


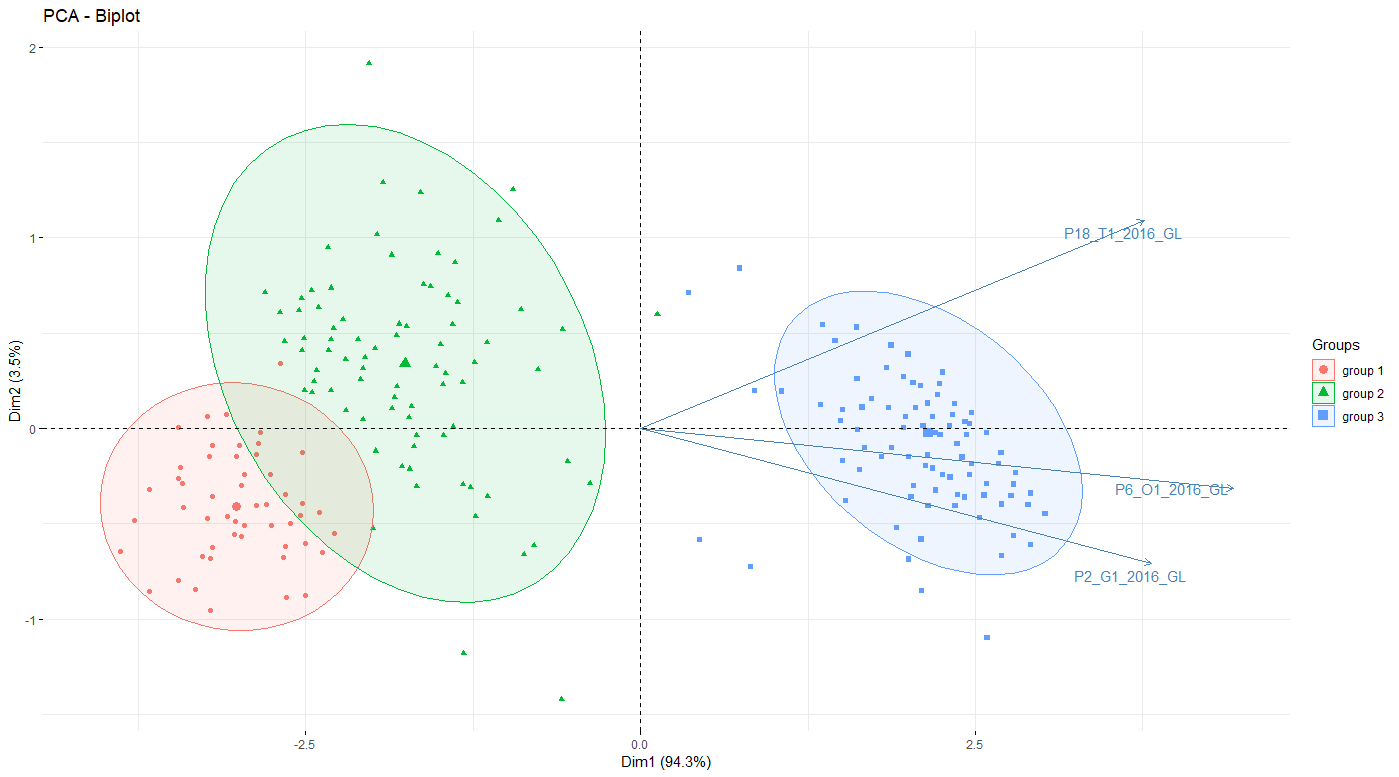


**Supplementary Figure 10**

Principal Component Analysis performed in the KxL population on P2, P6 and P18 resistance. The two first principal components explained 97.8% of the variance. The descendants could be divided in three groups: group 1 was composed of descendants resistant to P2, P6 and P18, group 2 was composed of descendants resistant to P2 and P6 but slightly susceptible to P18 and group 3 was composed of descendants susceptible to the four pathotypes.


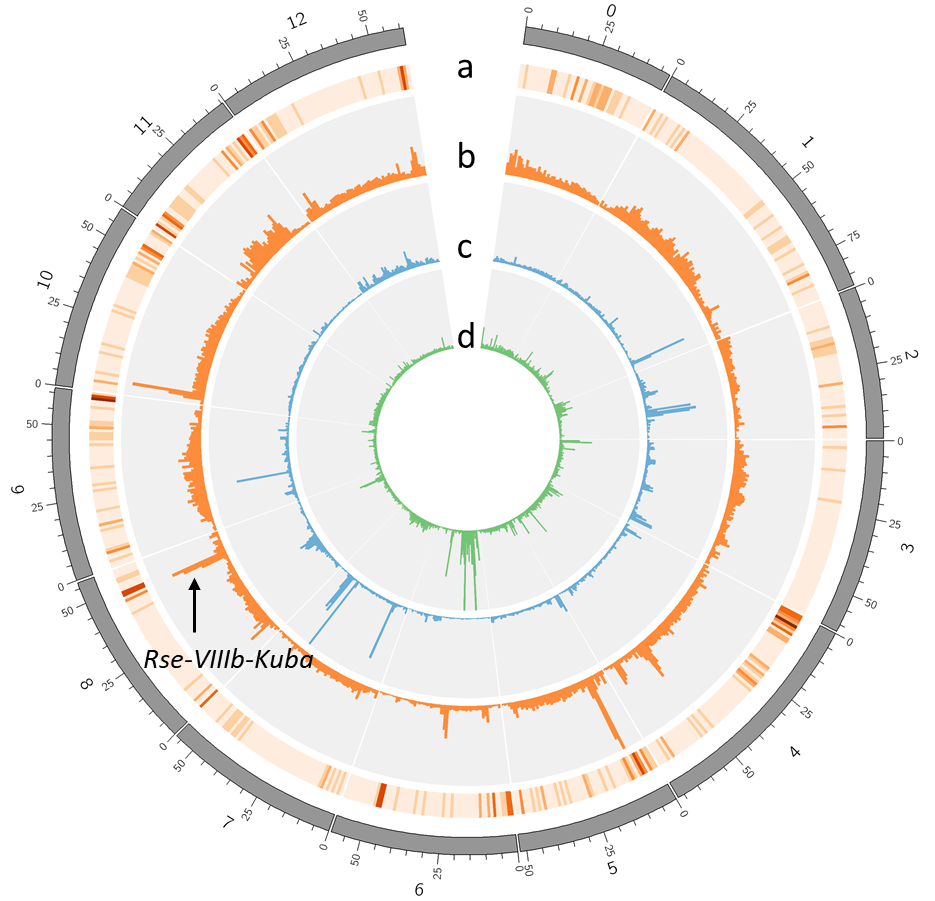


**Supplementary Figure 11**

Comparative Subread Sets Analysis performed in the KxL population. **a** Number of NLR genes per bin of 1 Mb from the potato reference genome DM v4.03 according to (Jupe *et al*., 2013). **b** KxL_RB specific *k*-mers inherited from Kuba mapped to the reference genome (y_max_ = 420%). **c** KxL_RB specific k-mers inherited from Ludmilla mapped to the reference genome (y_max_ = 452%). **d** KxL_RB specific *k*-mers inherited from both parents mapped to the reference genome (y_max_ = 444%). An arrow shows *Rse-VIIIb-Kuba* which is linked with P18 resistance.


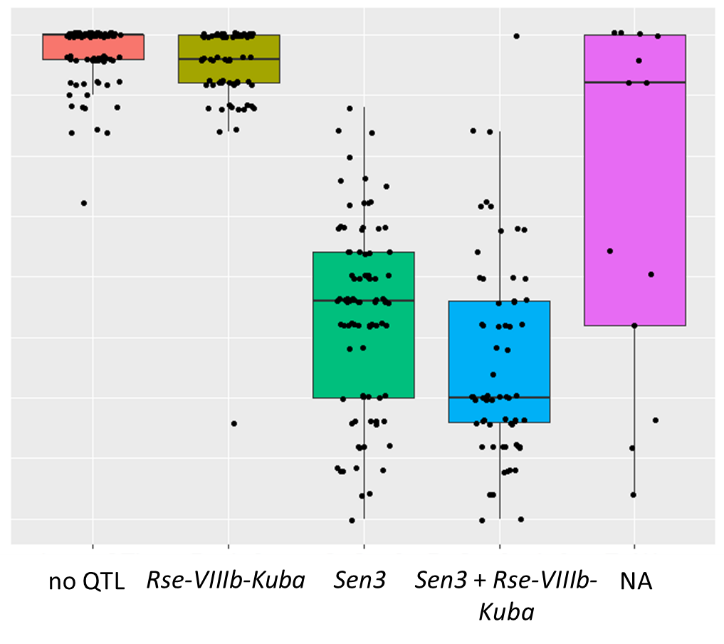


**Supplementary Figure 12**


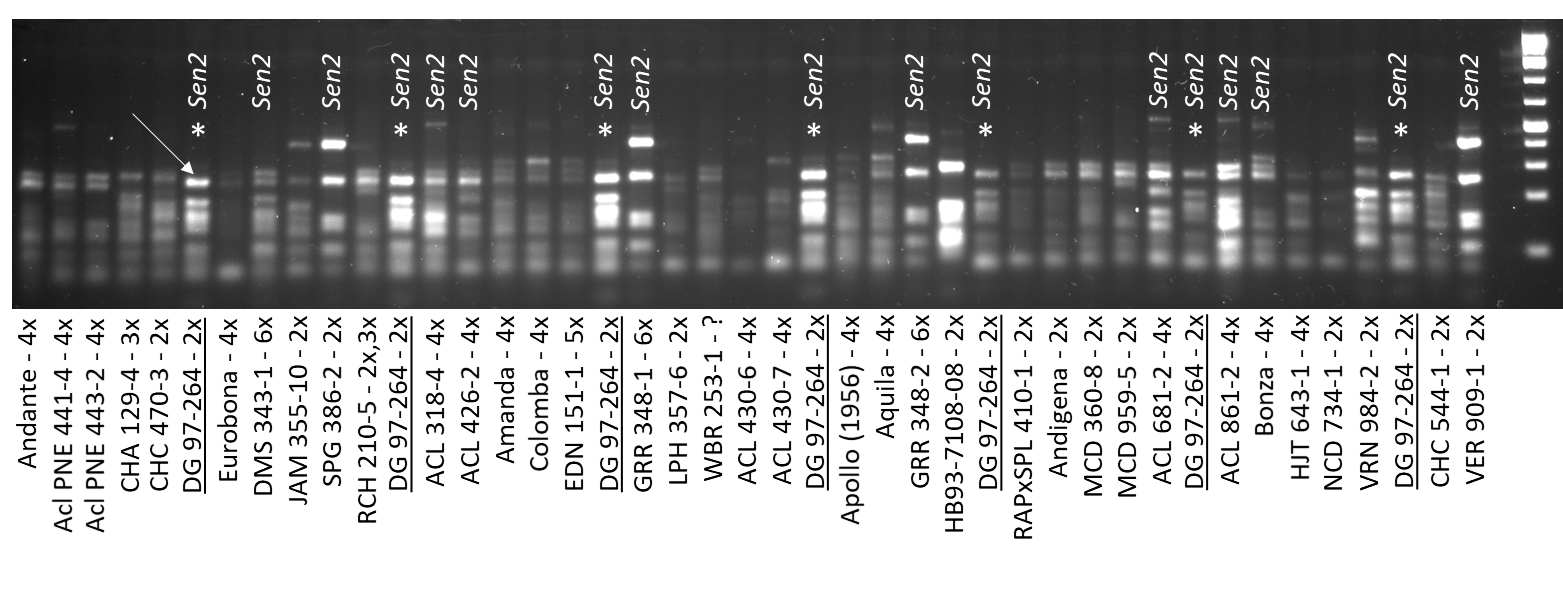
Effect of *Sen3* and of *Rse-VIIIb-Kuba* (chr08_44797542 and chr08_45178832) on P18 resistance in the KxL population.

**Supplementary Figure 13**

Gel picture of the digestion product of the CAPS marker linked with Sen2 for several varieties and accessions. The positive control (DG 97-264) is indicated by the mean of stars. To score Sen2 as being present or absent in the variety and Solanum panels, we looked at the presence of the 250 bp band (arrow), its intensity compared to DG 97-264 considering the ploidy level of the genotype, and the intensity of the smaller bands from DG 97-264. If the 1046 bp fragment is not amplified, or the 250 bp fragment (arrow) is absent, the genotype is scored negative for the marker. The genotypes scored as positive for Sen2 are indicated.

# Supplementary Tables

**Supplementary Table 1. Summary of the phenotypic assays performed in this study**

| Population | Method | Year | Place | Pathotypes | Isolates | # genotypes assessed | # tubers |
| --- | --- | --- | --- | --- | --- | --- | --- |
| AxV | Spieckermann | 2015 | HLB BV | P2 | HLB P2 (G1)-03-08 | 100 | 8 |
|  |  |  |  | P6 | HLB 6 (O1) 10-11 | 100 | 8 |
|  |  |  |  | P18 | HLB 18 (T1) 05-08 | 100 | 8 |
|  |  | 2016 | Averis seeds BV | P2 | HLB P2 (G1)-01-05 | 45 | 15 |
|  |  |  |  | P6 | HLB P6 (O1) 07-09 + HLB 14-13 | 75 | 15 |
|  |  |  |  | P18 | HLB P18 (T1) 09-11 + HLB 04-07 | 75 | 15 |
|  |  | 2017 |  | P2 | HLB P2 (G1)-03-08 | 28 | 15 |
|  |  |  |  | P6 | HLB 6 (O1) 10-11 + MB57 | 2 | 30 |
|  |  |  |  | P18 | HLB 18 (T1) 09-11 + HLB05-08 | 2 | 30 |
| AxD & KxA | Glynne-Lemmerzahl | 2015 | IHAR-PIB | P6 | JKI P6(O1)-2009 | 77 | 3 |
|  |  |  |  | P18 | JKI P18(T1)-2009 | 77 | 3 |
|  |  | 2016 |  | P2 | JKI P2(G1)-2009 | 75 | 6 |
|  |  |  |  | P6 | JKI P6(O1)-2009 | 46 | 6 |
|  |  | 2017 |  | P2 | JKI P2(G1)-2009 | 27 | 6 |
|  |  |  |  | P6 | JKI P6(O1)-2009 | 16 | 6 |
|  | Spieckermann | 2016 | HLB BV | P6* | HLB 6 (O1) 10-11* | 77* | 8* |
|  |  |  |  | P18 | HLB 18 (T1) 05-08 | 77 | 8 |
|  |  | 2017 |  | P6* | HLB P6 (O1) 10-11* | 58* | 12* |
|  |  |  |  | P18 | HLB P18 (T1) 05-08 | 27 | 12 |

* The results from these phenotypic tests were not considered for further analyses as the infection level was very low and led to a high number of escapes.
